# Association of the Comprehensive Care for Joint Replacement Model With Disparities in the Use of Total Hip and Total Knee Replacement

**DOI:** 10.1001/jamanetworkopen.2021.11858

**Published:** 2021-05-28

**Authors:** Caroline P. Thirukumaran, Yeunkyung Kim, Xueya Cai, Benjamin F. Ricciardi, Yue Li, Kevin A. Fiscella, Addisu Mesfin, Laurent G. Glance

**Affiliations:** 1 Department of Orthopaedics, University of Rochester, Rochester, New York; 2 Department of Public Health Sciences, University of Rochester, Rochester, New York; 3 Center for Musculoskeletal Research, University of Rochester, Rochester, New York; 4 Department of Biostatistics and Computational Biology, University of Rochester, Rochester, New York; 5 Department of Family Medicine, University of Rochester, Rochester, New York; 6 Center for Community Health and Prevention, University of Rochester, Rochester, New York; 7 Department of Anesthesiology and Perioperative Medicine, University of Rochester, Rochester, New York; 8 RAND Health, RAND, Boston, Massachusetts

## Abstract

**Importance:**

The Comprehensive Care for Joint Replacement (CJR) model is Medicare’s mandatory bundled payment reform to improve quality and spending for beneficiaries who need total hip replacement (THR) or total knee replacement (TKR), yet it does not account for sociodemographic risk factors such as race/ethnicity and income. Results of this study could be the basis for a Medicare payment reform that addresses inequities in joint replacement care.

**Objective:**

To examine the association of the CJR model with racial/ethnic and socioeconomic disparities in the use of elective THR and TKR among older Medicare beneficiaries after accounting for the population of patients who were at risk or eligible for these surgical procedures.

**Design, Setting, and Participants:**

This cohort study used the 2013 to 2017 national Medicare data and multivariable logistic regressions with triple-differences estimation. Medicare beneficiaries who were aged 65 to 99 years, entitled to Medicare, alive at the end of the calendar year, and residing either in the 67 metropolitan statistical areas (MSAs) mandated to participate in the CJR model or in the 104 control MSAs were identified. A subset of Medicare beneficiaries with a diagnosis of arthritis underwent THR or TKR. Data were analyzed from March to December 2020.

**Exposures:**

Implementation of the CJR model in 2016.

**Main Outcomes and Measures:**

Outcomes were separate binary indicators for whether a beneficiary underwent THR or TKR. Key independent variables were MSA treatment status, pre- or post-CJR model implementation phase, combination of race/ethnicity (non-Hispanic White, non-Hispanic Black, and Hispanic beneficiaries) and dual eligibility, and their interactions. Logistic regression models were used to control for patient characteristics, MSA fixed effects, and time trends.

**Results:**

The 2013 cohort included 4 447 205 Medicare beneficiaries, of which 2 025 357 (45.5%) resided in MSAs with the CJR model. The cohort’s mean (SD) age was 77.18 (7.95) years, and it was composed of 2 951 140 female (66.4%), 3 928 432 non-Hispanic White (88.3%), and 657 073 dually eligible (14.8%) beneficiaries. Before the CJR model implementation, rates were highest among non-Hispanic White non–dual-eligible beneficiaries at 1.25% (95% CI, 1.24%-1.26%) for THR use and 2.28% (95% CI, 2.26%-2.29%) for TKR use in MSAs with CJR model. Compared with MSAs without the CJR model and the analogous race/ethnicity and dual-eligibility group, the CJR model was associated with a 0.10 (95% CI, 0.05-0.15; *P* < .001) percentage-point increase in TKR use for non-Hispanic White non–dual-eligible beneficiaries, a 0.11 (95% CI, 0.004-0.21; *P* = .04) percentage-point increase for non-Hispanic White dual-eligible beneficiaries, a 0.15 (95% CI, −0.29 to −0.01; *P* = .04) percentage-point decrease for non-Hispanic Black non–dual-eligible beneficiaries, and a 0.18 (95% CI, −0.34 to −0.01; *P* = .03) percentage-point decrease for non-Hispanic Black dual-eligible beneficiaries. These CJR model–associated changes in TKR use were 0.25 (95% CI, −0.40 to −0.10; *P* = .001) percentage points lower for non-Hispanic Black non–dual-eligible beneficiaries and 0.27 (95% CI, −0.45 to −0.10; *P* = .002) percentage points lower for non-Hispanic Black dual-eligible beneficiaries compared with the model–associated changes for non-Hispanic White non–dual-eligible beneficiaries. No association was found between the CJR model and a widening of the THR use gap among race/ethnicity and dual eligibility groups.

**Conclusions and Relevance:**

Results of this study indicate that the CJR model was associated with a modest increase in the already substantial difference in TKR use among non-Hispanic Black vs non-Hispanic White beneficiaries; no difference was found for THR. These findings support the widespread concern that payment reform has the potential to exacerbate disparities in access to joint replacement care.

## Key Points

**Question:**

Is the Comprehensive Care for Joint Replacement (CJR) model associated with worsening of racial/ethnic and socioeconomic disparities in total hip replacement or total knee replacement use among older Medicare beneficiaries?

**Findings:**

In this cohort study of 4 447 205 Medicare beneficiaries, the CJR model was associated with an increase in total knee replacement use for non-Hispanic White beneficiaries and a decrease for non-Hispanic Black beneficiaries. The CJR model was also associated with widening of the gap in total knee replacement use among non-Hispanic White beneficiaries with non–dual eligibility for Medicaid compared with non-Hispanic Black beneficiaries with either non–dual eligibility or dual eligibility; no such widening of the gap was found for total hip replacement use.

**Meaning:**

The study found that the CJR model was associated with modest worsening of racial/ethnic and socioeconomic disparities in total knee replacement use, highlighting the need for payment reforms that incentivize the reduction of disparities.

## Teaser

This cohort study evaluates whether the implementation of a large-scale Medicare policy is associated with unequal use of and access to joint replacement care among older adults from various racial/ethnic groups and socioeconomic backgrounds.

## Introduction

The 2016 Comprehensive Care for Joint Replacement (CJR) model^1^ is Medicare’s mandatory bundled payment reform aimed at improving outcomes and reducing spending for older Medicare beneficiaries who need to undergo joint replacement (ie, total hip replacement [THR] or total knee replacement [TKR]). In the first 2 years of the CJR model implementation, more than 700 hospitals in 67 metropolitan statistical areas (MSAs) were mandated to participate. Under this model, hospitals are held accountable for the spending and quality of care during the inpatient stay for joint replacements and the 90-day postacute care period (episode). Hospitals are eligible to earn financial rewards if their spending for each 90-day episode is lower than a quality-adjusted target price, or hospitals are assessed penalties if their spending per episode is higher than this target price. Although the CJR model accounts for the patient’s clinical condition by setting different prices for Medicare Severity Diagnosis Related Groups and fractures, it does not account for sociodemographic risk factors, such as race/ethnicity and income.

The CJR model has led to modest reductions in joint replacement spending, with the decreases primarily associated with discharging patients to home instead of to postacute care facilities.^2^ However, the association of the CJR model with joint replacement use is mostly untested. In the absence of sociodemographic risk adjustment, hospitals may selectively avoid the use of joint replacement procedures as a treatment option for patients who are perceived to be at a greater risk of adverse outcomes and higher expenditures.^3,4,5,6^ This avoidance may reduce the opportunity for beneficiaries from racial/ethnic minority groups, especially those from lower socioeconomic strata (collectively known as socially disadvantaged beneficiaries), to undergo joint replacement, thereby exacerbating the persistent disparities.^7^ These concerns are supported by several factors, including complex health needs,^8^ the likelihood of postoperative complications and readmissions,^9,10^ and increased costs among socially disadvantaged patients; all of these factors are associated with higher spending and lower quality scores for hospitals. This CJR model–associated mechanism for worsening of use disparities adds to other mechanisms, such as patient preferences guided by inadequate information,^11,12^ uncertain expectations,^13^ and worse outcomes among family and friends,^14^ as well as clinician biases while recommending surgical procedures,^15,16^ and geographic factors associated with surgical access.^7^ These mechanisms have been found to be associated with the substantially lower joint replacement rate among Black beneficiaries, especially those with lower income compared with White beneficiaries.

Although studies that examined CJR model–associated changes in joint replacement use at the national level did not find changes in the clinical case mix,^2,17,18,19^ race/ethnicity or dual eligibility for Medicaid,^17,18,19^ or financial^2^ risk profile of Medicare patients admitted to hospitals, 2 studies identified decreases in surgical procedures for dual-eligible^2^ and Black Medicare beneficiaries.^20^ However, most of these studies focused on patients who were admitted to hospitals and did not account for the underlying population of patients who were at risk or eligible for these surgical procedures.^4^ Moreover, the national studies did not examine whether the association of the CJR model with surgical use for beneficiaries from various racial/ethnic groups was moderated by their socioeconomic status, and whether this association differed for hip vs knee replacements. Both socioeconomic status and type of surgical procedure are important patient selection pathways.

Given these gaps in the literature, the objective of the present study was to examine the association of the CJR model with racial/ethnic and socioeconomic (as measured by dual eligibility for Medicare and Medicaid) disparities in the use of elective THR and TKR among older Medicare beneficiaries, after accounting for the population of patients who were at risk or eligible for these surgical procedures. We hypothesized that the CJR model reduced the probability of THRs or TKRs for non-Hispanic Black, Hispanic, and dual-eligible Medicare beneficiaries residing in MSAs that implemented the CJR model. The results of this study could help lay the groundwork for a Medicare payment reform that successfully addresses inequities in joint replacement care.

## Methods

This cohort study was approved and granted a waiver of informed consent by the University of Rochester Research Subject Review Board because of the encrypted nature of the data. We followed the Strengthening the Reporting of Observational Studies in Epidemiology (STROBE) reporting guideline.^21^ Data were analyzed from March to December 2020.

### Data Sources and Study Cohort

We used the 2013 to 2017 Medicare Master Beneficiary Summary File (MBSF)–Base Segment^22^ enrollment files to identify fee-for-service Medicare beneficiaries who were aged 65 to 99 years, entitled to Medicare because of age eligibility, alive at the end of the calendar year, and residing either in 1 of the 67 MSAs that were mandated to participate in the CJR model or in 1 of the 104 MSAs identified as control that were not required to participate in the CJR model (eMethods 1 in the Supplement).^23^ These MSAs were randomly selected by Medicare.^24^ We used the US Bureau of Economic Analysis data to map the beneficiary county codes from the MBSFs to the MSAs.^25^ To limit the study cohort to beneficiaries who may be potentially at risk or eligible for THR or TKR, we used the 2013 to 2017 Medicare MBSF–Chronic Conditions Segment to include beneficiaries who met the claims criteria for arthritis (ie, osteoarthritis or rheumatoid arthritis).^26^

We used the 2013 to 2017 Medicare Provider Analysis and Review inpatient claims files^27^ to identify inpatient, short stays for fee-for-service Medicare beneficiaries residing in MSAs with the CJR model or MSAs without the CJR model. We used Medicare Severity Diagnosis Related Groups 469 and 470 to identify patients who had THR or TKR (additional criteria are described in eMethods 1 in the Supplement). Although the CJR model includes hospital stays for fractures, we excluded these stays from the study because hospitals are unlikely to selectively avoid joint replacement procedures as a treatment option for patients in urgent situations.^28^ We also excluded stays in hospitals that participated in the Bundled Payments for Care Improvement (BPCI) initiative (model 1, or risk-bearing phase of model 2 or 4).^29^ We constructed annual beneficiary-level binary indicators for THRs and TKRs.

We merged the enrollment and inpatient claims files to limit the analytic cohort to fee-for-service non-Hispanic White, non-Hispanic Black, and Hispanic Medicare beneficiaries with arthritis (<0.1% of the patients who had THR or TKR did not have an arthritis diagnosis) and who resided in MSAs with the CJR model or MSAs without the CJR model. Beneficiaries who underwent THR or TKR but were excluded from the inpatient claims cohort because they did not meet the inclusion criteria (eg, THRs or TKRs in BPCI hospitals or joint replacements for fractures) were also excluded from the enrollment files to prevent an underestimation of the magnitude of any potential associations. The final analytic cohort comprised 23 239 775 beneficiary-year observations from 2013 to 2017 for 9 074 191 unique beneficiaries, of whom 242 646 had THR and 455 257 had TKR.

### Key Variables

#### Outcomes

The outcomes were separate binary indicators for whether a beneficiary underwent THR or TKR. Beneficiaries with arthritis residing in MSAs with the CJR model or MSAs without the CJR model who did not meet the THR or TKR inclusion criteria were classified as being at risk or eligible for THR or TKR but did not undergo these procedures (eMethods 1 in the Supplement).

#### Key Independent Variables and Covariates 

To examine whether the association of the CJR model with the probability of THRs or TKRs varied across non-Hispanic White, non-Hispanic Black, Hispanic, and dual-eligible beneficiaries, we used the following key independent variables: a binary indicator for MSAs with the CJR model or MSAs without the CJR model, a binary indicator for pre-CJR model (2013-2015) or post-CJR model implementation (2017), a categorical indicator for the race/ethnicity and dual-eligibility combination (non-Hispanic White dual-eligible or non–dual-eligible, non-Hispanic Black dual-eligible or non–dual-eligible, and Hispanic dual-eligible or non–dual-eligible), and relevant interactions (eg, 3-way interaction between the type of the MSA, the CJR model implementation phase, and the race/ethnicity and dual eligibility of the beneficiary) (eMethods 2 in the Supplement). We excluded the 2016 data from the main multivariable analysis because the CJR model was implemented in April 2016, which precluded the classification of beneficiaries at risk of THRs or TKRs into the pre- or post-CJR model cohorts.

We identified race/ethnicity from the indicator in the MBSF, which originated from the US Social Security Administration records, and beneficiaries who met state-reported dual-eligibility criteria for 12 months in a year were identified as potentially dually eligible for Medicaid. We chose dual eligibility as a proxy for socioeconomic status because of its strong dependence on having low income.^7,30,31^ We controlled for the calendar year and patient-level risk factors, such as age, sex, and binary indicators for 24 chronic conditions (eTable 1 in the Supplement), in a multivariable analysis.

### Statistical Analysis

#### Descriptive and Multivariable Analyses

We used χ^2^ and Kruskal-Wallis tests to assess the differences in the distribution of key characteristics of patients and MSAs. We also plotted annual trends in THR or TKR use by race/ethnicity dual eligibility and MSA treatment status.

We constructed beneficiary year–level multivariable logistic regression models (separate for THRs and TKRs) with MSA fixed effects and Huber-White robust or sandwich estimators of variance to examine whether beneficiaries from racial/ethnic minority groups with dual eligibility were more or less likely to undergo THR or TKR after CJR model implementation vs beneficiaries in MSAs without the CJR model. We used the triple-differences approach to isolate the independent association of the CJR model with THR or TKR use.^32,33^ We addressed the violation of the parallel trends assumptions for the triple-differences models by including interactions of the year with MSAs with the CJR model or MSAs without the CJR model and race/ethnicity dual-eligibility indicators (eMethods 2 in the Supplement).^34,35,36^ We obtained the adjusted estimated probabilities from these regression models and used tests for linear combinations to examine the hypotheses. Using MSA-level weights, we followed the Lewin Group’s methods to account for the selection probabilities of MSAs with the CJR model vs MSAs without the CJR model (eMethods 3 in the Supplement).^23^

All statistical analyses were performed with Stata/MP, version 16.1, for Unix (StataCorp LLC). A 2-tailed *P* < .05 was considered to be statistically significant.

#### Sensitivity Analyses

We conducted several sensitivity analyses (eMethods 2 in the Supplement). First, we used the cohort of 75 MSAs (intention-to-treat analysis) that were originally mandated to participate in the CJR model.^2^ Second, we used the Research Triangle Institute indicator to ascertain patient race/ethnicity.^37^ Third, we redefined dual eligibility using the Medicare entitlement or buy-in code.^38^ Fourth, we included data from 2016 in the post-CJR model implementation period. Fifth, we refined the definition of elective surgical procedures by applying the algorithm that Medicare uses to identify the elective THR or TKR cohort while computing risk-standardized complication and readmission rates.^39^ Sixth, we estimated the differential association by generating interactions with race/ethnicity (or dual eligibility) and the MSAs with the CJR model or MSAs without the CJR model and the CJR phase indicators. Seventh, to examine whether beneficiaries from racial/ethnic minority groups in MSAs with the CJR model may have been directed to undergo THRs or TKRs elsewhere, we estimated multivariable logistic regressions that modeled a binary indicator of whether patients underwent surgery in their residence MSA as the outcome and the CJR model implementation phase, the race/ethnicity dual-eligibility indicator, and their interaction as key independent variables.

## Results

### Descriptive Analysis

For 2013, the cohort included 4 447 205 Medicare beneficiaries with arthritis, of which 2 025 357 (45.5%) resided in MSAs with the CJR model (Table 1). The cohort’s mean (SD) age was 77.18 (7.95) years and included 2 951 140 female (66.4%), 3 928 432 non-Hispanic White (88.3%), and 657 073 dually eligible (14.8%) beneficiaries (Table 1). The distribution of race/ethnicity, dual eligibility, and chronic conditions was significantly different between MSAs with the CJR model and MSAs without the CJR model. The MSAs with the CJR model compared with MSAs without the CJR model had a lower rate of THR (1.0% vs 1.1%; *P* < .001) and TKR (2.0% vs 2.2%; *P* < .001) (Table 1). eTables 1-3 in the Supplement present additional descriptive statistics.

**Table 1.** Descriptive Statistics of Medicare Beneficiaries With Rheumatoid Arthritis or Osteoarthritis Residing in Metropolitan Statistical Areas (MSAs) With or Without the Comprehensive Care for Joint Replacement (CJR) Model, 2013

Variable	MSAs without CJR model	MSAs with CJR model	Total	*P* value^a^
No. of patients^b^	2 421 848 (54.5)	2 025 357 (45.5)	4 447 205	
Age, mean (SD), y	76.98 (7.91)	77.43 (7.99)	77.18 (7.95)	<.001
Sex				
Female, No. (%)	1 599 361 (66.0)	1 351 779 (66.7)	2 951 140 (66.4)	<.001
Male, No. (%)	822 487 (34.0)	673 578 (33.3)	1 496 065 (33.6)	
Race/ethnicity, No. (%)				
Non-Hispanic White	2 152 536 (88.9)	1 775 896 (87.7)	3 928 432 (88.3)	<.001
Non-Hispanic Black	232 064 (9.6)	174 683 (8.6)	406 747 (9.2)	
Hispanic	37 248 (1.5)	74 778 (3.7)	112 026 (2.5)	
Dual eligibility for Medicaid and Medicare, No. (%)	306 429 (12.7)	350 644 (17.3)	657 073 (14.8)	<.001
Race/ethnicity by dual eligibility, No. (%)				
Non-Hispanic White NDE beneficiaries	1 947 596 (80.4)	1 550 369 (76.6)	3 497 965 (78.7)	<.001
Non-Hispanic White DE beneficiaries	204 940 (8.5)	225 527 (11.1)	430 467 (9.7)	
Non-Hispanic Black NDE beneficiaries	154 508 (6.4)	110 898 (5.5)	265 406 (5.97)	
Non-Hispanic Black DE beneficiaries	77 556 (3.2)	63 785 (3.2)	141 341 (3.2)	
Hispanic NDE beneficiaries	13 315 (0.6)	13 446 (0.7)	26 761 (0.6)	
Hispanic DE beneficiaries	23 933 (1.0)	61 332 (3.0)	85 265 (1.9)	
No. of chronic conditions by race/ethnicity, mean (SD)^c^				
Non-Hispanic White	4.37 (2.59)	4.59 (2.66)	4.47 (2.63)	<.001
Non-Hispanic Black	4.94 (2.65)	4.92 (2.66)	4.94 (2.66)	.01
Hispanic	4.74 (2.70)	5.52 (2.72)	5.26 (2.74)	<.001
No. of chronic conditions by dual eligibility, mean (SD)^c^				
Non–dual eligibility	4.26 (2.54)	4.39 (2.58)	4.32 (2.56)	<.001
Dual eligibility	5.55 (2.76)	5.90 (2.76)	5.74 (2.76)	<.001
Type of surgery, No. (%)^d^				
THR	25 682 (1.1)	20 425 (1.0)	46 107 (1.0)	<.001
TKR	53 977 (2.2)	40 574 (2.0)	94 551 (2.1)	<.001
MSAs^e^				
No.	104	67	171	
Total population in 1000s, median (IQR)	439 (223-961)	467 (234-1757)	446 (225-1134)	.56
% of Population >65 y, mean (SD)	15.26 (4.83)	14.46 (3.87)	14.94 (4.48)	.27
% of Females, mean (SD)	50.81 (0.85)	51.01 (0.82)	50.89 (0.84)	.14
% of High school graduates, mean (SD)	87.79 (4.22)	88.01 (3.61)	87.88 (3.98)	.96
Income, mean (SD), $	68 675 (12 787)	69 275 (11 479)	68 912 (12 257)	.66

Abbreviations: DE, dual-eligible; IQR, interquartile range; NDE, non–dual eligible; THR, total hip replacement; TKR, total knee replacement.

^a^ *P* values were calculated with Kruskal-Wallis tests (for continuous variables) or χ^2^ tests (for categorical variables). Kruskal-Wallis and χ^2^ tested for the differences in the distribution of characteristics across MSAs with vs without the CJR model.

^b^ Data were from the 2013 Medicare Master Beneficiary Summary File–Base Segment and Chronic Conditions Segment.

^c^ Mean of 24 chronic conditions included in the Medicare Master Beneficiary Summary File–Chronic Conditions Segment.

^d^ Data were from the 2013 Medicare Provider Analysis and Review file.

^e^ Data were from the American Community Survey File.

**Figure 1.**
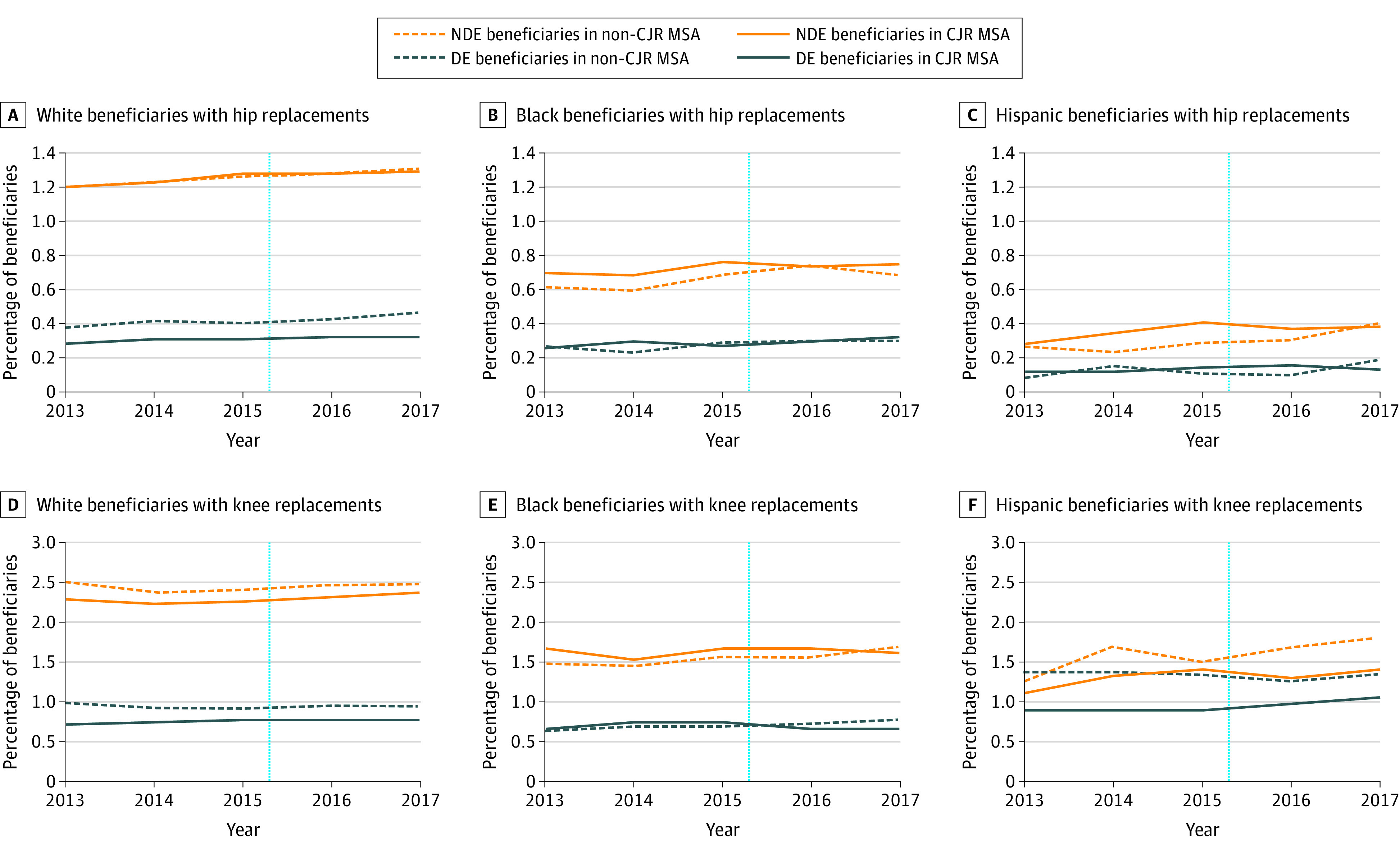
Unadjusted Trends in the Percentage of Medicare Beneficiaries Who Underwent Hip and Knee Replacements in the Metropolitan Statistical Areas (MSAs) With Comprehensive Care for Joint Replacement (CJR) and Without CJR Model Data show the analysis of the 2013 to 2017 Medicare Master Beneficiary Summary File (Base and Chronic Conditions Segment) and Medicare Provider Analysis and Review File. The year markings on the x-axis represent the end of the respective year. The dotted vertical line represents the date of CJR model implementation in April 2016. DE indicates dual-eligible; NDE, non–dual-eligible.

Before CJR model implementation, the THR and TKR rates were highest among non-Hispanic White non–dual-eligible beneficiaries, with 1.25% (95% CI, 1.24%-1.26%) for THR and 2.28% (95% CI, 2.26%-2.29%) for TKR in MSAs with the CJR model compared with 1.24% (95% CI, 1.23%-1.25%) for THR and 2.44% (95% CI, 2.43%-2.45%) for TKR in MSAs without the CJR model (Figure 1 and Table 2). The THR rate was 0.31% (95% CI, 0.30%-0.32%) for non-Hispanic White, 0.28% (95% CI, 0.26%-0.31%) for non-Hispanic Black, and 0.13% (95% CI, 0.11%-0.15%) for Hispanic dual-eligible beneficiaries in MSAs with the CJR model. The TKR rate was 0.77% (95% CI, 0.75%-0.80%) for non-Hispanic White, 0.74% (95% CI, 0.70%- 0.78%) for non-Hispanic Black, and 0.90% (95% CI, 0.86%-0.95%) for Hispanic dual-eligible beneficiaries. Although TKR use increased by 4.39% for non-Hispanic White non–dual-eligible beneficiaries and by 5.19% for non-Hispanic White dual-eligible beneficiaries in MSAs with the CJR model implementation, TKR use decreased by 1.21% for non-Hispanic Black non–dual-eligible beneficiaries and by 8.11% for non-Hispanic Black dual-eligible beneficiaries in these MSAs (eTable 4 in the Supplement).

**Table 2.** Association of the Comprehensive Care for Joint Replacement (CJR) Model With the Probabilities of Total Hip or Total Knee Replacement, 2013-2017^a^

Variable	Unadjusted probabilities, % (N = 18 403 141 observations; 171 MSAs)						Adjusted probabilities, % (N = 18 403 141 observations; 171 MSAs)	
	MSAs with CJR model^b^			MSAs without CJR model^c^			Percentage point change in MSAs with vs without CJR model (95% CI)^d^	Percentage point change with respect to White NDE beneficiaries (95% CI)^e^
	Before implementation (95% CI)	After implementation (95% CI)	Percentage point change (95% CI)	Before implementation (95% CI)	After implementation (95% CI)	Percentage point change (95% CI)		
**THR**								
Race/ethnicity by dual eligibility								
Non-Hispanic White NDE beneficiaries	1.25 (1.24 to 1.26)	1.31 (1.30 to 1.33)	0.07 (0.05 to 0.09)^f^	1.24 (1.23 to 1.25)	1.32 (1.30 to 1.33)	0.08 (0.06 to 0.09)^f^	0.05 (0.01 to 0.09)^g^	1 [Reference]
Non-Hispanic White DE beneficiaries	0.31 (0.30 to 0.32)	0.33 (0.31 to 0.36)	0.02 (−0.004 to 0.05)	0.40 (0.39 to 0.42)	0.47 (0.44 to 0.50)	0.07 (0.03 to 0.10)^f^	−0.02 (−0.08 to 0.05)	−0.06 (−0.14 to 0.01)
Non-Hispanic Black NDE beneficiaries	0.73 (0.70 to 0.76)	0.76 (0.71 to 0.80)	0.03 (−0.03 to 0.08)	0.64 (0.62 to 0.66)	0.70 (0.66 to 0.74)	0.06 (0.01 to 0.11)^g^	−0.002 (−0.10 to 0.10)	−0.05 (−0.16 to 0.06)
Non-Hispanic Black DE beneficiaries	0.28 (0.26 to 0.31)	0.33 (0.29 to 0.38)	0.05 (−0.002 to 0.10)	0.27 (0.25 to 0.29)	0.31 (0.27 to 0.35)	0.04 (−0.01 to 0.09)	0.06 (−0.05 to 0.17)	0.01 (−0.11 to 0.13)
Hispanic NDE beneficiaries	0.35 (0.29 to 0.41)	0.39 (0.29 to 0.50)	0.05 (−0.07 to 0.17)	0.26 (0.21 to 0.31)	0.40 (0.30 to 0.51)	0.14 (0.02 to 0.26)^**g**^	0.05 (−0.24 to 0.33)	−0.001 (−0.29 to 0.29)
Hispanic DE beneficiaries	0.13 (0.11 to 0.15)	0.14 (0.11 to 0.17)	0.01 (−0.03 to 0.05)	0.11 (0.08 to 0.13)	0.18 (0.13 to 0.24)	0.08 (0.01 to 0.14)^**g**^	−0.09 (−0.24 to 0.05)	−0.14 (−0.29 to 0.01)
**TKR**								
Race/ethnicity by dual eligibility								
Non-Hispanic White NDE beneficiaries	2.28 (2.26 to 2.29)	2.38 (2.36 to 2.41)	0.11 (0.08 to 0.14)^f^	2.44 (2.43 to 2.45)	2.47 (2.45 to 2.49)	0.03 (0.003 to 0.05)^**g**^	0.10 (0.05 to 0.15)^f^	1 [Reference]
Non-Hispanic White DE beneficiaries	0.77 (0.75 to 0.80)	0.81 (0.77 to 0.85)	0.04 (−0.01 to 0.08)	0.96 (0.94 to 0.99)	0.98 (0.94 to 1.03)	0.02 (−0.03 to 0.07)	0.11 (0.004 to 0.21)^**g**^	0.01 (−0.10 to 0.12)
Non-Hispanic Black NDE beneficiaries	1.65 (1.61 to 1.69)	1.63 (1.56 to 1.70)	−0.02 (−0.10 to 0.06)	1.52 (1.49 to 1.56)	1.68 (1.62 to 1.74)	0.15 (0.08 to 0.22)^f^	−0.15 (−0.29 to −0.01)^**g**^	−0.25 (−0.40 to −0.10)^h^
Non-Hispanic Black DE beneficiaries	0.74 (0.70 to 0.78)	0.68 (0.61 to 0.75)	−0.06 (−0.14 to 0.01)	0.70 (0.66 to 0.73)	0.79 (0.72 to 0.85)	0.09 (0.01 to 0.16)^**g**^	−0.18 (−0.34 to −0.01)^**g**^	−0.27 (−0.45 to −0.10)^h^
Hispanic NDE beneficiaries	1.30 (1.18 to 1.41)	1.41 (1.21 to 1.61)	0.12 (−0.11 to 0.35)	1.52 (1.40 to 1.64)	1.82 (1.60 to 2.05)	0.31 (0.05 to 0.56)^**g**^	0.05 (−0.55 to 0.66)	−0.04 (−0.65 to 0.57)
Hispanic DE beneficiaries	0.90 (0.86 to 0.95)	1.06 (0.96 to 1.15)	0.15 (0.05 to 0.26)^h^	1.38 (1.29 to 1.47)	1.37 (1.22 to 1.52)	−0.01 (−0.19 to 0.16)	0.32 (−0.001 to 0.64)	0.23 (−0.10 to 0.55)

Abbreviations: DE, dual-eligible; MSA, metropolitan statistical area; NDE, non–dual-eligible; THR, total hip replacement; TKR, total knee replacement.

^a^ Adjusted probabilities (expressed in percentages) from patient-level multivariable logistic regression models with robust or sandwich estimators of variance. The regression models assessed the association of the CJR model with the probability of surgical procedures for each race/ethnicity dual-eligibility group (vs non-Hispanic White NDE beneficiaries) in MSAs with vs without the CJR model. The regression models controlled for age, sex, comorbidities, calendar year (and interactions with indicators in MSAs with the CJR model and with race/ethnicity dual-eligibility indicator), MSA fixed effects, and MSA weights. The analysis excluded data from 2016 because the CJR model was introduced in April 2016, which precluded the classification of Medicare beneficiaries into pre- and post-CJR model cohorts. For the adjusted columns, the probabilities and changes in probabilities were obtained using Stata margins and lincom commands (StataCorp LLC).

^b^ Presents the unadjusted probabilities (expressed in percentages) of surgical procedures in MSAs with CJR model implementation and the percentage point differences with CJR model implementation.

^c^ Presents the unadjusted probabilities (expressed in percentages) of surgical procedures in MSAs without CJR model implementation and the percentage point differences with CJR model implementation.

^d^ Presents the percentage point differences in the probability of surgical procedures for each race/ethnicity dual-eligibility group in MSAs with vs without CJR model implementation.

^e^ Presents the percentage point differences in the probability of surgical procedures for each race/ethnicity dual-eligibility group (vs non-Hispanic White NDE beneficiaries) in MSAs with vs without CJR model implementation (triple difference).

^f^ *P* < .001.

^g^ *P* < .05.

^h^ *P* < .01.

### Multivariable Analysis

The results of the parallel trends analysis are presented in eTable 5 in the Supplement. After controlling for patient characteristics and MSA fixed effects, the CJR model was associated with a 0.05 (95% CI, 0.01-0.09; *P* = .02) percentage-point increase in THR use for non-Hispanic White non–dual-eligible beneficiaries in MSAs with the CJR model compared with MSAs without the CJR model (Table 2). However, the change in THR use with CJR model implementation was not significant across race/ethnicity dual-eligibility categories (Table 2). For example, the THR use rate for non-Hispanic Black non–dual-eligible beneficiaries was 0.05 (95% CI, −0.16 to 0.06; *P* = .37) percentage points lower in MSAs with the CJR model as compared with the rate for non-Hispanic White non–dual-eligible beneficiaries.

For the probability of undergoing TKR, the CJR model implementation was associated with a 0.10 (95% CI, 0.05-0.15; *P* < .001) percentage-point increase for non-Hispanic White non–dual-eligible beneficiaries, a 0.11 (95% CI, 0.004-0.21; *P* = .04) percentage-point increase for non-Hispanic White dual-eligible beneficiaries, a 0.15 (95% CI, −0.29 to −0.01; *P* = .04) percentage-point decrease for non-Hispanic Black non–dual-eligible beneficiaries, and a 0.18 (95% CI, −0.34 to −0.01; *P* = .03) percentage-point decrease for non-Hispanic Black dual-eligible beneficiaries in MSAs with the CJR model compared with the same categories in MSAs without the CJR model. The lower probability of use translated to 163 fewer surgical procedures (6.8%) among non-Hispanic Black non–dual-eligible beneficiaries and to 134 fewer procedures (18.7%) for non-Hispanic Black dual-eligible beneficiaries, after the CJR model implementation (eTable 4 in the Supplement).

The CJR model implementation was associated with a 0.25 (95% CI, −0.40 to −0.10; *P* = .001) percentage-point decrease in TKR probability for non-Hispanic Black non–dual-eligible beneficiaries and a 0.27 (95% CI, −0.45 to −0.10; *P* = .002) percentage-point decrease for non-Hispanic Black dual-eligible beneficiaries compared with non-Hispanic White non–dual-eligible beneficiaries, widening the TKR use gap between non-Hispanic White non–dual-eligible beneficiaries and Black (non–dual-eligible and dual-eligible) beneficiaries. Given that non-Hispanic White non–dual-eligible beneficiaries were twice as likely to undergo TKR as non-Hispanic Black dual-eligible beneficiaries before the CJR model implementation (eTable 6 in the Supplement), the decreases in TKR use associated with the implementation need to be considered given the preexisting disparities.

**Figure 2.**
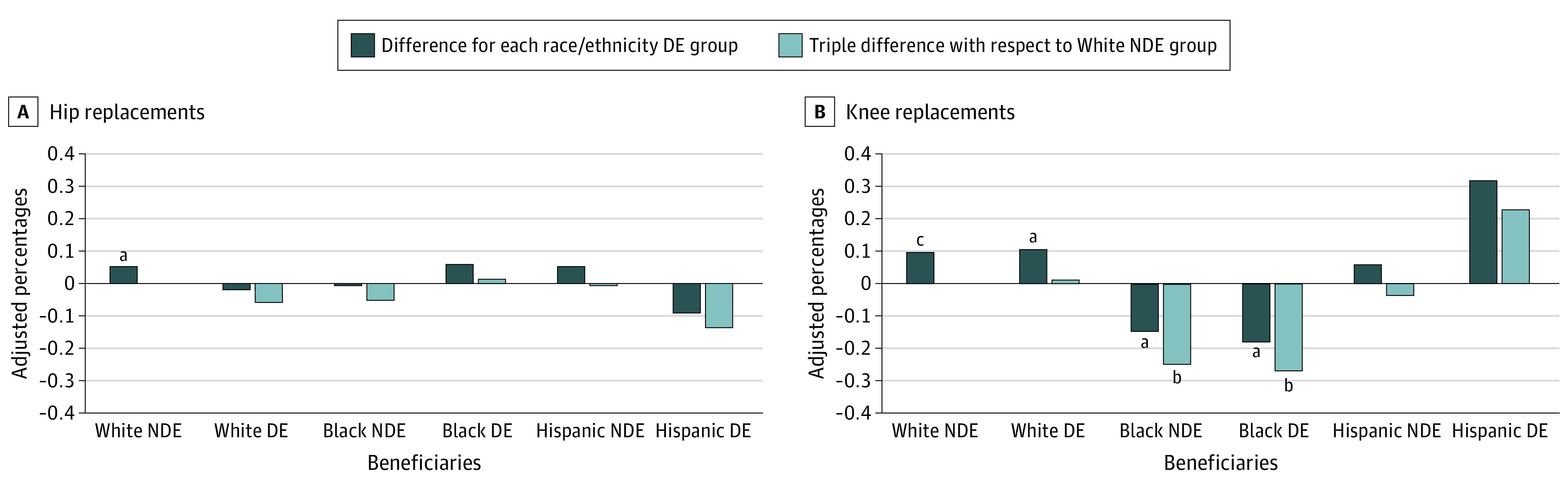
Differences in Adjusted Percentages of Hip and Knee Replacement Use With Comprehensive Care for Joint Replacement (CJR) Model Implementation Differences in adjusted percentages were derived from patient-level multivariable logistic regression models with robust or sandwich estimators of variance (Table 2). The difference for each race/ethnicity dual-eligible (DE) group key represents the percentage point difference in the probability of surgical procedures in the metropolitan statistical areas (MSAs) with CJR model implementation vs those MSAs without CJR. The triple difference (vs non-Hispanic White non–dual-eligible [NDE] beneficiaries) key represents the percentage point difference in the probability of procedures for each race/ethnicity DE group (vs non-Hispanic White NDE group) in MSAs with CJR model implementation vs MSAs without CJR model. ^a^*P* < .05. ^b^*P* < .01. ^c^*P* < .001.

We did not find changes in THR or TKR use that were associated with the CJR model implementation for Hispanic beneficiaries. The changes in adjusted probabilities are presented in Figure 2, and regression estimates are presented in eTable 6 in the Supplement.

### Sensitivity Analysis

The findings from sensitivity analyses were generally consistent with the main results (eTables 7-9 in the Supplement). We did not find evidence that the CJR model was associated with changes in the probability that beneficiaries from racial/ethnic minority groups would undergo surgical procedures in their residence MSA except for non-Hispanic White non–dual-eligible and dual-eligible beneficiaries (eTable 9 in the Supplement).

## Discussion

Reducing health care disparities has long been a priority in the US. This analysis of national Medicare data from 2013 to 2017, however, showed that the CJR model, one of the few large-scale health policy interventions using random assignment, was associated with modest reductions in TKR use for non-Hispanic Black Medicare beneficiaries with both dual and non–dual eligibility, in comparison to non-Hispanic White non–dual-eligible beneficiaries. This worsening of the disparities is important because it is superimposed on substantial and persistent preexisting inequality in TKR use (non-Hispanic White non–dual-eligible beneficiaries were twice as likely to undergo TKR as non-Hispanic Black dual-eligible beneficiaries before the CJR model implementation). In contrast, the CJR model was not associated with an increase in THR disparities. We also did not find evidence of changes in THR or TKR use that were associated with the CJR model for Hispanic beneficiaries vs for non-Hispanic White non–dual-eligible beneficiaries. By focusing on patients with arthritis, including patients who were at risk for surgical procedures so that we could account for underlying trends,^4^ controlling for time trends before the CJR model implementation, and controlling for chronic conditions, we generated rigorous evidence of the association of the CJR model with disparities in joint replacement use. A focus on both race/ethnicity and dual eligibility of the patient and a focus on THR and TKR separately tested the moderating attribute of socioeconomic status in access to joint replacement care.^3^

We believe that this cohort study fills a gap in the bundled payment and CJR model literature, particularly the association of Medicare payment reforms with access to care for socially disadvantaged beneficiaries. Most of the previous national studies on patient selection in the CJR model did not find a CJR model–associated change in the risk profile (as measured by clinical case mix,^2,17,18,19^ race/ethnicity dual eligibility,^17,18,19^ or risk of high overall spending^2^) of patients who underwent joint replacement. Two exceptions were the study by Barnett et al,^2^ which found 0.4% fewer joint replacement for dual-eligible beneficiaries, and a study by Kim et al,^20^ which found 0.64 per 1000 fewer joint replacements for Black beneficiaries. However, methodological considerations differentiated the present study and findings from those of previous CJR model–focused studies. Most previous studies examined a cohort of patients who were admitted to hospitals for joint replacement and did not explicitly account for the underlying population of patients who were eligible for these surgical procedures. Although these approaches tested changes in the clinical and spending profiles of patients who had surgical procedures, they did not account for beneficiaries who may have potentially benefitted from these procedures but were denied the opportunity to undergo these procedures. Moreover, previous studies examined the THR and TKR cohorts as a single group, thereby not accounting for the heterogeneity in these cohorts. Studies that examined patient selection in the closely aligned voluntary BPCI initiative found variable results^40^ that ranged from no evidence of changes in the clinical complexity of patients^41^ to evidence of decreases in previous health care use and case mix^42,43^ to an increase in the case complexity of patients who had spinal fusion.^44^ Thus, little is known about the association of bundled payments with racial/ethnic and income profiles of patients who underwent joint replacement, after accounting for those in need of these surgical procedures. To our knowledge, this study is the first to address these limitations and to address gaps in knowledge.

We believe the findings of the present study provide empirical evidence to address previous concerns regarding patient selection.^3,4,5,6^ In the absence of risk adjustment of the financial targets or quality metrics for sociodemographic factors under the CJR model,^45^ hospitals may be more likely to avoid joint replacement procedures as a treatment option for non-Hispanic Black beneficiaries because they bear a disproportionately higher burden of comorbidities than non-Hispanic White beneficiaries (Table 1) and are at a greater risk of poor surgical outcomes and higher spending.^9,10,46,47^ This rationale for unfavorable selection is further supported by concerns that the investments made in quality improvement in preparation for payment reforms and the resulting capabilities may motivate hospitals to increase their case volumes with perceived healthier patients, thereby leaving out beneficiaries from racial/ethnic minority groups and increasing the existing disparities.^5^ Furthermore, the absence of having to consider sociodemographic risk adjustment places an increased burden on safety net hospitals,^48,49,50^ and these hospitals may be particularly cautious in selecting socially vulnerable patients for surgical procedures. Potential explanations for CJR model–associated disparities in TKRs and not in THRs are likely to be a greater need for institutional rehabilitation after TKRs, especially for beneficiaries from racial/ethnic minority groups,^51,52^ higher adverse events,^53^ and longer recovery.^54^ Moreover, the absence of evidence of adverse selection among Hispanic beneficiaries was reassuring, but further research is needed to advance understanding.

In addition to the CJR model, other payment reforms, such as the BPCI, the BPCI Advanced,^55^ and the Medicare Shared Savings Program^56^ (which similarly do not adequately adjust for sociodemographic risk), may reduce joint replacement use in socially disadvantaged beneficiaries. This lack of risk adjustment may create perverse incentives for hospitals to avoid joint replacement use in socially complex patients.^57^ The CJR model implementation was associated with a modest worsening of disparities in TKR use, but the study found that the CJR model was associated with not only failure to improve but also worse racial/ethnic equity in TKR use. We believe that Medicare could create metrics that incentivize hospitals to reduce disparities. At a minimum, Medicare could give hospitals credit for caring for socially vulnerable individuals by adjusting for sociodemographic risk and directly incentivizing equity.^45^ One policy initiative could be the reporting of access among racial/ethnic minority groups and other relevant metrics as a part of program evaluation. These study findings thus highlight the need for further studies of the association between current Medicare payment reforms and inequalities in joint replacement use.

### Limitations

This study has several limitations. First, the data sets used did not include data on patient preferences or clinical risk. The differences in these factors may be partly responsible for the baseline differences in THR and TKR use across groups. However, these preferences were unlikely to have had differential changes (for race/ethnicity, dual eligibility, and MSA treatment status) over the study period. Second, to account for the patient population at risk for THRs or TKRs,^4^ we used a claims-based diagnosis of osteoarthritis and rheumatoid arthritis. Although this measure may include beneficiaries with arthritis of other joints, such as the ankle or shoulder, this distribution was unlikely to have had differential changes over time. Moreover, identifying patients with end-stage arthritis or the type of arthritis would have been beneficial. However, because we used administrative data, the severity and type of arthritis could not be reliably ascertained. Third, a portion of the at-risk population, especially non-Hispanic White beneficiaries, may have previously undergone THR or TKR and hence may not have been truly at risk for these surgical procedures. Although the distribution of previous procedures may be different at baseline for various groups, we do not expect this distribution to change over time. Fourth, the CJR model design was changed from a fully mandated to a partly mandated and partly voluntary program in 2018. Because of this change in design, this study was limited to examining the CJR model only until 2017 and could not examine longer-term outcomes.

## Conclusions

The CJR model may have been associated with worsening of racial/ethnic and socioeconomic disparities in TKR use. Given the disparities in joint replacement use that existed even before the CJR model implementation, Medicare could adjust for sociodemographic risk and create metrics that incentivize hospitals to reduce disparities. These steps may help ensure that older adults of all races/ethnicities and socioeconomic strata have equal opportunities to access joint replacement care.

## Notes

Supplement. **eMethods 1.** Cohort determination criteria **eMethods 2.** Key variables, model estimation, and sensitivity analysis **eMethods 3.** Weighting strategy to account for MSA selection probability **eTable 1.** Chronic conditions for Medicare beneficiaries residing in CJR and non-CJR MSAs with a diagnosis of rheumatoid arthritis/osteoarthritis in 2013 **eTable 2.** Descriptive statistics for Medicare beneficiaries residing in CJR and non-CJR MSAs with a diagnosis of rheumatoid arthritis/osteoarthritis who underwent hip replacements in 2013 **eTable 3.** Descriptive statistics for Medicare beneficiaries residing in CJR and non-CJR MSAs with a diagnosis of osteoarthritis/rheumatoid arthritis who underwent knee replacements in 2013 **eTable 4.** Changes in knee replacements with CJR implementation **eTable 5.** Adjusted odds ratios for parallel trends test from multivariable models examining the odds of undergoing hip and knee replacement surgeries before the Comprehensive Care for Joint Replacement model was implemented (2013-2015) **eTable 6.** Full model estimates (adjusted odds ratios) from multivariable models examining the odds of undergoing hip or knee replacement surgeries before and after the Comprehensive Care for Joint Replacement model was implemented (2013-2017) **eTable 7.** Sensitivity analyses (1-6) - the Comprehensive Care for Joint Replacement model’s association with the probability of undergoing hip replacements with respect to White non-dual-eligible beneficiaries (2013-2017) **eTable 8.** Sensitivity analyses (1-6) - the Comprehensive Care for Joint Replacement model’s association with the probability of undergoing knee replacements with respect to White non-dual-eligible beneficiaries (2013-2017) **eTable 9.** Sensitivity analysis (7) – the Comprehensive Care for Joint Replacement model’s effect on the probability of undergoing surgery in the MSA of residence eReferences
