# Supplementary material for: Association of the Comprehensive Care for Joint Replacement Model With Disparities in the Use of Total Hip and Total Knee Replacement
Source: JAMA Netw Open. 2021 May 28;4(5):e2111858. doi: 10.1001/jamanetworkopen.2021.11858 (PMC8164097; doi:10.1001/jamanetworkopen.2021.11858)
Supplement: Supplement. — eMethods 1. Cohort determination criteria eMethods 2. Key variables, model estimation, and sensitivity analysis eMethods 3. Weighting strategy to account for MSA selection probability eTable 1. Chronic conditions for Medicare beneficiaries residing in CJR and non-CJR MSAs with a diagnosis of rheumatoid arthritis/osteoarthritis in 2013 eTable 2. Descriptive statistics for Medicare beneficiaries residing in CJR and non-CJR MSAs with a diagnosis of rheumatoid arthritis/osteoarthritis who underwent hip replacements in 2013 eTable 3. Descriptive statistics for Medicare beneficiaries residing in CJR and non-CJR MSAs with a diagnosis of osteoarthritis/rheumatoid arthritis who underwent knee replacements in 2013 eTable 4. Changes in knee replacements with CJR implementation eTable 5. Adjusted odds ratios for parallel trends test from multivariable models examining the odds of undergoing hip and knee replacement surgeries before the Comprehensive Care for Joint Replacement model was implemented (2013-2015) eTable 6. Full model estimates (adjusted odds ratios) from multivariable models examining the odds of undergoing hip or knee replacement surgeries before and after the Comprehensive Care for Joint Replacement model was implemented (2013-2017) eTable 7. Sensitivity analyses (1-6) - the Comprehensive Care for Joint Replacement model’s association with the probability of undergoing hip replacements with respect to White non-dual-eligible beneficiaries (2013-2017) eTable 8. Sensitivity analyses (1-6) - the Comprehensive Care for Joint Replacement model’s association with the probability of undergoing knee replacements with respect to White non-dual-eligible beneficiaries (2013-2017) eTable 9. Sensitivity analysis (7) – the Comprehensive Care for Joint Replacement model’s effect on the probability of undergoing surgery in the MSA of residence eReferences [file jamanetwopen-e2111858-s001.pdf]

Supplementary Online Content

Thirukumaran CP, Kim Y, Cai X, et al. Association of the Comprehensive Care for Joint Replacement model with disparities in the use of total hip and total knee replacement. *JAMA Netw Open*. 2021;4(5):e2111858. doi:10.1001/jamanetworkopen.2021.11858

eMethods 1. Cohort determination criteria

eMethods 2. Key variables, model estimation, and sensitivity analysis

eMethods 3. Weighting strategy to account for MSA selection probability

eTable 1. Chronic conditions for Medicare beneficiaries residing in CJR and non-CJR MSAs with a diagnosis of rheumatoid arthritis/osteoarthritis in 2013

eTable 2. Descriptive statistics for Medicare beneficiaries residing in CJR and non-CJR MSAs with a diagnosis of rheumatoid arthritis/osteoarthritis who underwent hip replacements in 2013

eTable 3. Descriptive statistics for Medicare beneficiaries residing in CJR and non-CJR MSAs with a diagnosis of osteoarthritis/rheumatoid arthritis who underwent knee replacements in 2013

eTable 4. Changes in knee replacements with CJR implementation

eTable 5. Adjusted odds ratios for parallel trends test from multivariable models examining the odds of undergoing hip and knee replacement surgeries before the Comprehensive Care for Joint Replacement model was implemented (2013-2015)

eTable 6. Full model estimates (adjusted odds ratios) from multivariable models examining the odds of undergoing hip or knee replacement surgeries before and after the Comprehensive Care for Joint Replacement model was implemented (2013-2017)

eTable 7. Sensitivity analyses (1-6) - the Comprehensive Care for Joint Replacement model's association with the probability of undergoing hip replacements with respect to White non-dual-eligible beneficiaries (2013-2017)

eTable 8. Sensitivity analyses (1-6) - the Comprehensive Care for Joint Replacement model's association with the probability of undergoing knee replacements with respect to White non-dual-eligible beneficiaries (2013-2017)

eTable 9. Sensitivity analysis (7) – the Comprehensive Care for Joint Replacement model's effect on the probability of undergoing surgery in the MSA of residence

eReferences

This supplementary material has been provided by the authors to give readers additional information about their work.

© 2021 Thirukumaran CP et al. *JAMA Network Open.*

eMethods 1. Cohort determination criteria

<u>Inclusion criteria using the Medicare Master Beneficiary Summary (MBSF) File (2013-2017)</u>

- Beneficiaries residing in CJR and non-CJR MSAs
- Fee-for-service beneficiaries identified as those with fee-for-service Medicare coverage for 12 months in the year
- Beneficiaries between 65-99 years of age
- Old age and not end-stage renal disease as a reason for Medicare entitlement
- Beneficiaries who were alive at the end of the calendar year
- Beneficiaries with no missing data for race, dual-eligibility, or sex
- Beneficiaries are attributed to the MSA determined using their zip code at the end of the year.

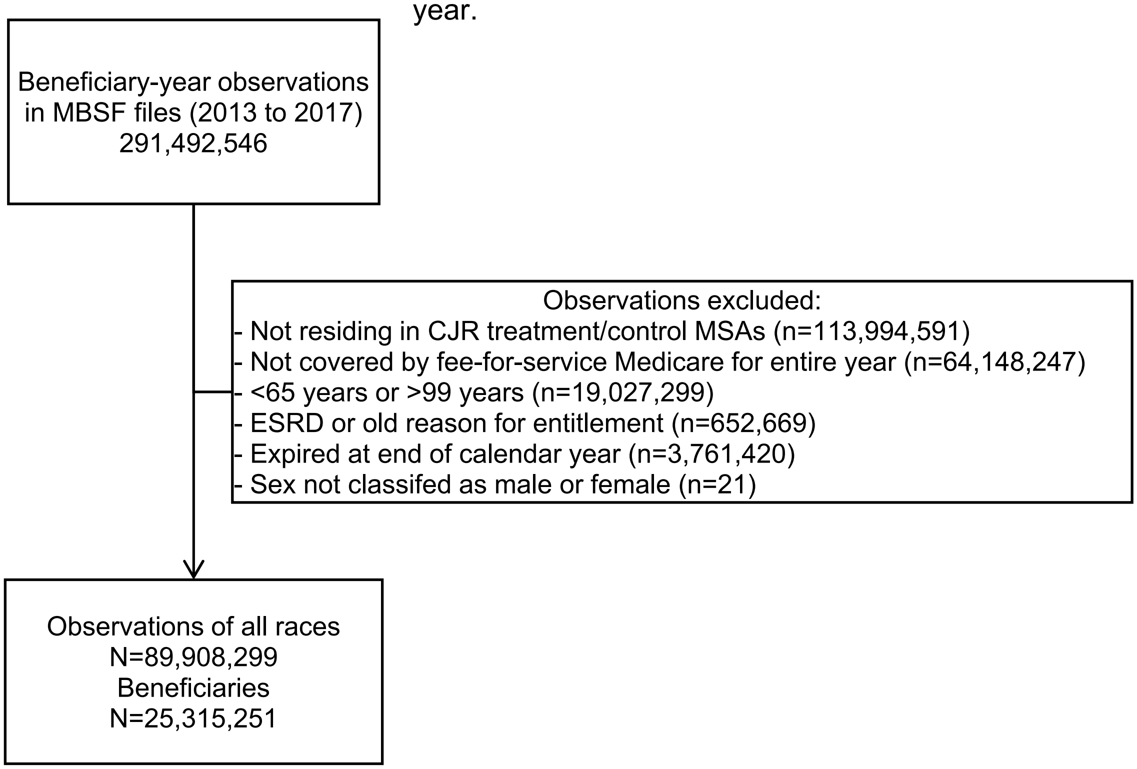

© 2021 Thirukumaran CP et al. *JAMA Network Open.*

Inclusion criteria using the Medicare Provider Analysis and Review file (MedPAR) (2013-2017)

- Medical Severity Diagnosis Related Group (MS-DRG) codes 469 and 470
- Meet MBSF inclusion criteria listed above
- Short, inpatient stays
- Hospitals reimbursed by the Inpatient Prospective Payment System
- Medicare as the primary payor
- Alive during the 90-day episode
- Non-fracture related, elective stays
- Enrolled in Parts A and B of Medicare at the time of admission
- Hospitals not participating in Model 1 or the risk-bearing phase of Models 2 and 3 of the Bundled Payments for Care Improvement initiative
- Inpatient stays for total hip replacement and total knee replacement
- Non-duplicate inpatient stays
- Qualifying stays that do not occur during the 90-day episode of a previous qualifying stay

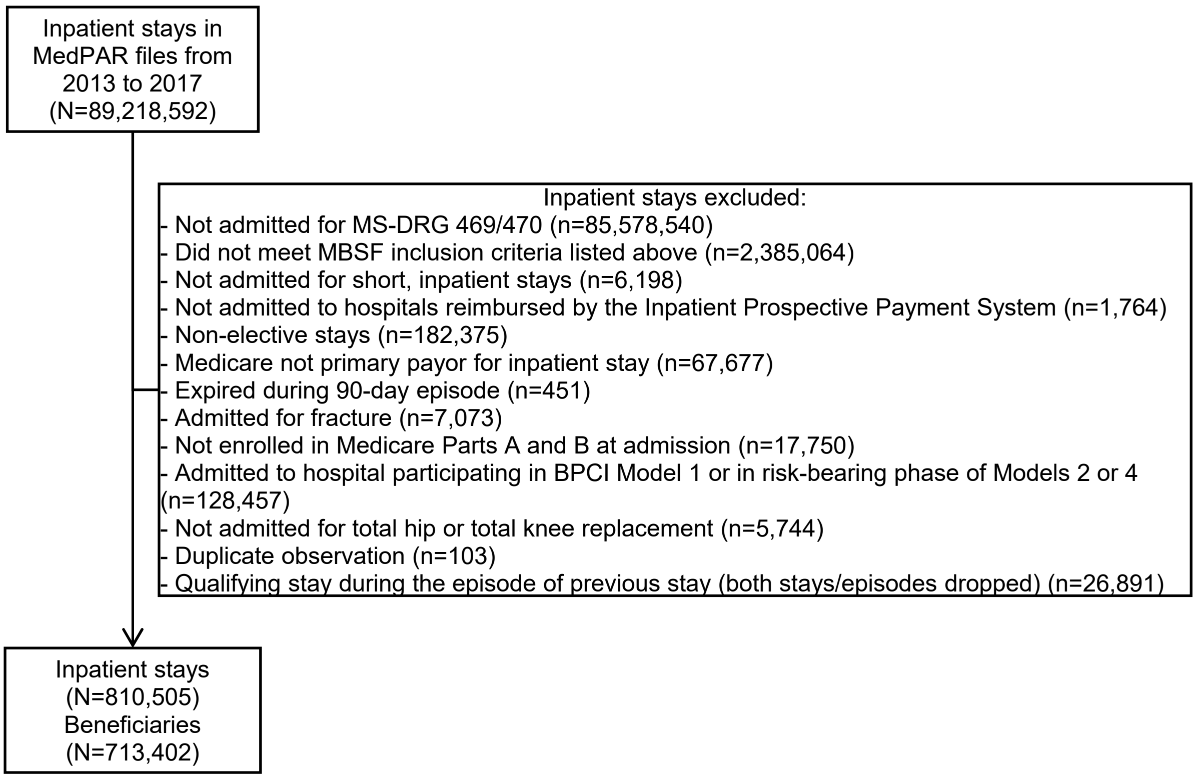

© 2021 Thirukumaran CP et al. *JAMA Network Open.*

We used the 2013-2017 Medicare Provider Analysis and Review (MedPAR) inpatient claims files[1] to identify inpatient, short-stays for fee-for-service Medicare beneficiaries residing in the CJR and non-CJR MSAs. We used Medical Severity-Diagnosis Related Groups (MS-DRG) 469 and 470 to identify patients undergoing total hip/knee replacements. We excluded non-elective stays, stays for whom Medicare was not the primary payor, if patients expired during the anchor stay or in the 90 days after discharge from the stay (deaths among patients undergoing surgeries in the last quarter of 2017 were identified using 2018 files), and if patients were not enrolled in Medicare Parts A and B at the time of inpatient admission. If a qualifying stay was initiated in a previous qualifying episode, we excluded both episodes.[2] While CJR includes stays for fractures, we excluded these stays from our study because surgeons are unlikely to discriminate between patients in urgent situations.[3] We also excluded stays from hospitals participating in the Bundled Payments for Care Improvement (BPCI) initiative (Model 1 or risk-bearing phase of Models 2 or 4),[4] and those admitted to hospitals not reimbursed by the Inpatient Prospective Payment System. We constructed annual beneficiary-level binary indicators for hip/knee replacements.

© 2021 Thirukumaran CP et al. *JAMA Network Open.*

Inclusion criteria from merging the MBSF and MedPAR files (2013-2017)

Note: The files were merged at the beneficiary-level. Two binary indicators (one for hip and one for knee replacements) were created to represent whether a beneficiary had one or more qualifying stay during the year.

- Beneficiaries with joint replacements that meet CJR criteria or beneficiaries who did not undergo any joint replacements
- White, Black, and Hispanic beneficiaries
- Beneficiaries with a diagnosis of osteoarthritis / rheumatoid arthritis
- No hip fracture claims

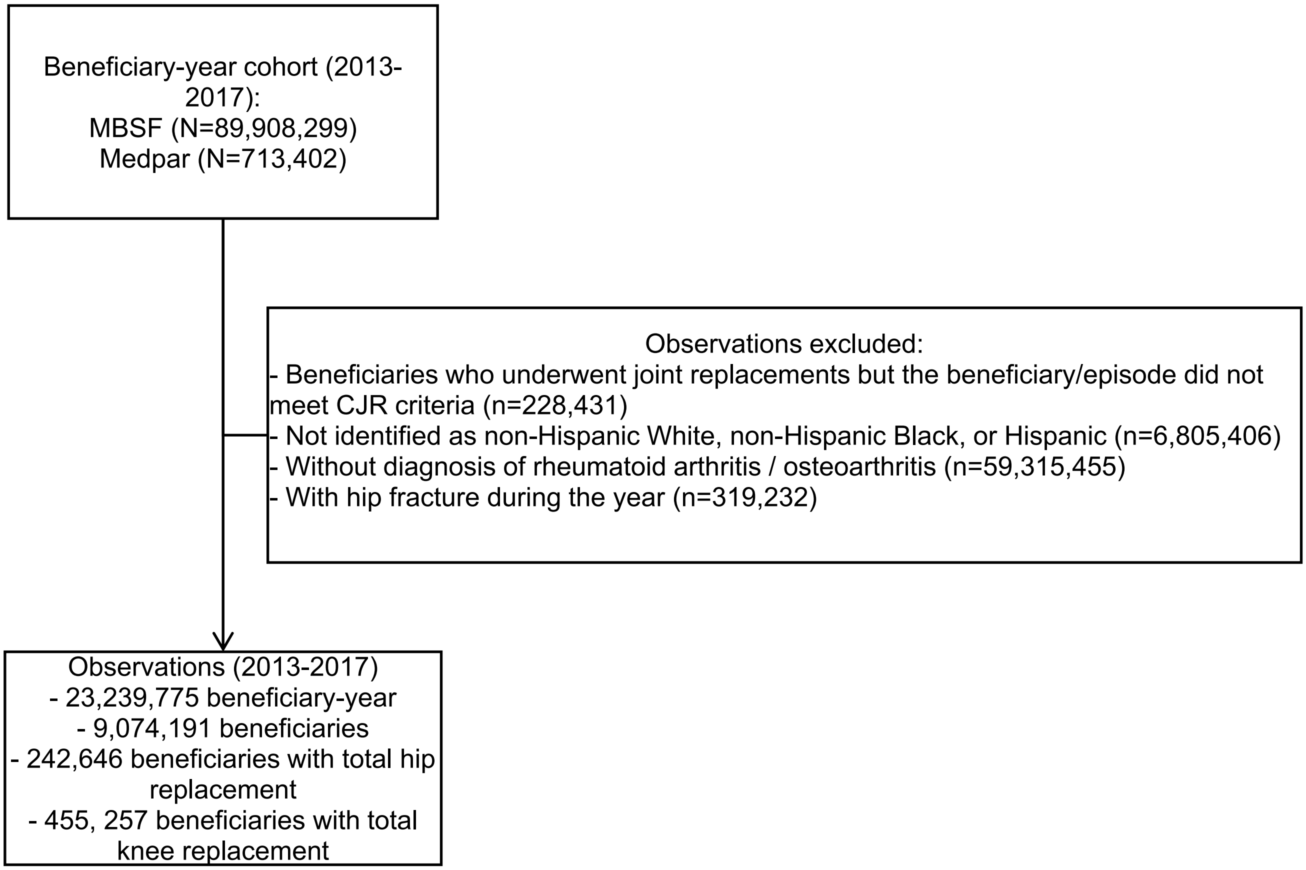

© 2021 Thirukumaran CP et al. *JAMA Network Open.*

eMethods 2. Key variables, model estimation, and sensitivity analysis

Key independent variables: Race and dual-eligibility

We identified beneficiary race from the MBSF enrollment files in which the data originates from the Social Security Administration records. We identified dual-eligibility using the state-reported dual-eligible status code (the dual_elgble_mos_num variable in the MBSF files).[5] Beneficiaries who met dual-eligibility criteria for twelve months in the year were classified as dual-eligible beneficiaries.

Test for parallel trends assumption for triple differences models

To assess whether the trends in the use of joint replacements for Medicare beneficiaries from the various race-dual-eligibility groups were parallel in the period before the CJR was implemented (parallel trends assumption for the triple differences models), we estimated the following models (separate for hip and knee replacements). The data for these models was limited to 2013-2015 (pre-CJR period).

$$f\left[E\left(Y_{pmt}\right)\right] = \beta_0 + \beta_1 \times Race\_Dual_{pmt} + \beta_2 \times CJR_m + \beta_3 \times Race\_Dual_{pmt} \times CJR_m + \beta_4 \times Year_t$$
$$+ \ \beta_5 \times Year_t \times Race\_Dual_{pmt} + \beta_6 \times Year_t \times CJR_m$$
$$+ \beta_7 \times Year_t \times Race\_Dual_{pmt} \times CJR_m + \ \beta_9 \times X_{pmt} + \beta_{10} MSA_m$$

Y_{pmt} (hip_dum/knee_dum): Binary indicator of whether patient p residing in MSA m in year t underwent hip (or knee) replacement

$f(.)$: Log link function

CJR_m (cjr_dum): Binary indicator of whether MSA m was a CJR or non-CJR MSA

$Year_t$ (year): Categorical indicator of the year

X_{pmt} ($patient_covariates): Vector of patient covariates include age, sex, and 24 chronic conditions identified from the MBSF-CC file

© 2021 Thirukumaran CP et al. *JAMA Network Open.*

MSA_m (msa): Fixed effects of MSA represented using a categorical indicator

$Race\_Dual_{pmt}$ (race_dual): A categorical indicator of race and dual-eligibility (White non-dual-eligible, White dual-eligible, Black non-dual-eligible, Black dual-eligible, Hispanic non-dual-eligible, and Hispanic dual-eligible)

We used the Wald test to separately test for the significance of β_5, β_6, and β_7. A p-value <0.05 on the test for any of the three estimates in a model represented a violation of the parallel trends assumption.

Results of the parallel trends tests for triple differences models

The parallel trends tests were significant for both hip and knee replacements.

Model estimation for triple differences models

We estimated the following triple differences models (separate for hip and knee replacements) to assess the differential effect of the CJR. The triple differences approach includes the estimation of three differences: First, the difference between the pre- and post-CJR rates for each race-dual-eligibility group in CJR and non-CJR MSAs (difference 1). Second, the difference in difference 1 between CJR and non-CJR MSAs for each race-dual-eligibility group (difference 2). Third, the difference in difference 2 between each race-dual-eligibility group (except for White non-dual-eligible beneficiaries) and White non-dual-eligible beneficiaries (difference 3).

Because of the violation of the parallel trends assumption, we included interactions of $Year_t$ with $Race\_Dual_{pmt}$ and CJR_m to account for the differential trends in the pre-CJR period.[6,7] This specification is similar to Comparative Interrupted Time Series models which do not impose the parallel trends assumption.[8]

© 2021 Thirukumaran CP et al. *JAMA Network Open.*

$$
\begin{aligned}
f\left[E\left(Y_{pmt}\right)\right] = {} & \beta_0 + \beta_1 \times Race\_Dual_{pmt} + \beta_2 \times CJR_m + \beta_3 \times Race\_Dual_{pmt} \times CJR_m + \beta_4 \times Year_t \\
& + \beta_5 \times Post_t + \beta_6 \times Post_t \times Race\_Dual_{pmt} + \beta_7 \times Post_t \times CJR_m \\
& + \beta_8 \times Post_t \times Race\_Dual_{pmt} \times CJR_m + \beta_9 \times X_{pmt} + \beta_{10} MSA_m \\
& + \beta_{11} \times Year_t \times Race\_Dual_{pmt} + \beta_{12} \times Year_t \times CJR_m \\
& + \beta_{13} \times Year_t \times Race\_Dual_{pmt} \times CJR_m
\end{aligned}
$$

$Post_t$ is a binary indicator for the CJR implementation phase (Years 2013-2015=0, year 2017=1). The interpretation of other terms is similar to those described previously. During model estimation $Year_t$=2017 is excluded from the model estimates due to collinearity with the $Post_t$=1 category. β_8 is the triple differences estimate for CJR's association with disparities for each race/ethnic-dual-eligibility group as compared to White non-dual-eligible beneficiaries.

We used Stata/MP 16.1 for Unix to estimate the models. We used Stata's margins and lincom commands to test the study hypotheses.

Sensitivity analysis

We checked for the robustness of the main findings using the following strategies. *First,* we re-estimated the main models using the cohort of 75 MSAs (intention-to-treat analysis) that were originally mandated to participate in the CJR.[2] We used 121 control MSAs that originally met CJR inclusion criteria but were not selected for participation through randomization. *Second,* because the Research Triangle Institute (RTI) indicator for race is more sensitive for the identification of Hispanic beneficiaries, we re-estimated the main models using the RTI indicator. This indicator uses a combination of names and geographic locations to improve the identification of Hispanic and Asians/Pacific Islander beneficiaries in comparison to their identification from the Social Security Administration records.[9, 10] *Third,* we redefined dual-eligibility using the Medicare entitlement/buy-in code, which identifies dual-eligible beneficiaries as those with state buy-in for Parts A or B or both of Medicare.[5] *Fourth*, the intent to implement

the CJR was published in July 2015, and the rule was finalized in November 2015. Because changes in joint replacement use patterns may have set-in in advance of the April 2016 start date, we re-estimated the main models by including data from 2016 in the post-CJR period. *Fifth*, to refine the identification of elective surgeries, we used Medicare's definition for elective joint replacements which was designed for identifying the cohort for risk-standardized readmission and complication rates.[11, 12] This approach uses ICD-9-PCS and ICD-10-PCS codes instead of MS-DRGs (used by the CJR) for identification of joint replacements. *Sixth*, to examine the effects of race and dual-eligibility separately, we estimated the differential effect models by interacting race (instead of race-dual-eligibility combination) with the CJR/non-CJR MSA and CJR phase indicators. We conducted similar analysis by interacting dual-eligibility with CJR/non-CJR MSA and CJR phase indicators. *Finally*, to examine whether racial minorities may have been directed to undergo joint replacements in MSAs not participating in the CJR, we estimated multivariable logistic regression models that modeled a binary indicator of whether the patient underwent surgery in his/her residence MSA. This analysis was limited to beneficiaries residing in CJR MSAs. The key independent variables were the CJR phase, the race-dual-eligibility indicator, and the interactions between these variables.

© 2021 Thirukumaran CP et al. *JAMA Network Open.*

eMethods 3. Weighting strategy to account for MSA selection probability

We used the method by the Lewin Group to account for an MSA's probability of selection into the treatment (CJR MSAs) or control (non-CJR MSAs) group.[13] In this approach, the CJR MSAs were assigned a weight of 1 and the non-CJR MSAs were assigned weights to represent the CJR MSAs. These weights for the non-CJR MSAs were obtained by dividing the number of CJR MSAs in each of the 8 strata (constructed by the CMS using quartiles of pre-period episode spending and whether the MSA had above or below median population) by the number of non-CJR MSAs in that stratum. These weights were then used in our regression models using Stata's pweight option which are "weights that denote the inverse of the probability that the observation is included because of the sampling design."[14]

Exhibit E-9: CMS' stratified random sample of CJR MSAs and analytic weights

MSA population	MSA sampling stratum	MSA average episode payment	# MSAs eligible for sampling	CJR sample			Control group sample		
				# CJR MSAs	Proportion of MSAs selected for CJR	CJR weight	# Control group MSAs	Proportion of MSAs in the control group	Control group weight
Less than median population	1	Lowest quartile	25	8	32.0%	1.0	17	68.0%	8/17
	2	2nd lowest quartile	18	6	33.3%	1.0	12	66.7%	6/12
	3	3rd lowest quartile	19	8	42.1%	1.0	11	57.9%	8/11
	4	Highest quartile	22	11	50.0%	1.0	11	50.0%	11/11
More than median population	5	Lowest quartile	15	5	33.3%	1.0	10	66.7%	5/10
	6	2nd lowest quartile	28	10	35.7%	1.0	18	64.3%	10/18
	7	3rd lowest quartile	22	9	40.9%	1.0	13	59.1%	9/13
	8	Highest quartile	22	10	45.5%	1.0	12	54.5%	10/12
		Total	171	67			104		

Source: Lewin analysis of the Medicare Program Comprehensive Care for Joint Replacement Payment Model for Acute Care Hospitals Furnishing Lower Extremity Joint Replacement Services; A Final Rule by the Centers for Medicare & Medicaid Services, 80 FR 73273 (November 24, 2015) (codified at 42 CFR 510).

Note: MSA = metropolitan statistical area.

Source: Lewin Group, CMS Comprehensive Care for Joint Replacements Model: Performance Year 2 Evaluation Report – Appendices, June 2019, Page 29.

© 2021 Thirukumaran CP et al. *JAMA Network Open.*

eTable 1. Chronic conditions for Medicare beneficiaries residing in CJR and non-CJR MSAs with a diagnosis of rheumatoid arthritis/osteoarthritis in 2013

	Non-CJR MSAs	CJR MSAs	Total	p-value[c]
**Patients[a]**				
N	2,421,848	2,025,357	4,447,205	
Chronic Conditions[b]: % (95% CI)				
Acute myocardial infarction	0.87 (0.86, 0.88)	0.87 (0.86, 0.88)	0.87 (0.86, 0.88)	0.95
Alzheimer's disease and its related dementias	14.84 (14.79, 14.88)	16.17 (16.12, 16.22)	15.44 (15.41, 15.48)	<0.001
Anemia	32.08 (32.02, 32.14)	37.53 (37.47, 37.60)	34.56 (34.52, 34.61)	<0.001
Asthma	7.46 (7.43, 7.49)	7.66 (7.62, 7.69)	7.55 (7.53, 7.58)	<0.001
Atrial fibrillation	12.07 (12.03, 12.11)	12.15 (12.10, 12.19)	12.10 (12.07, 12.14)	0.015
Benign prostatic hyperplasia	8.44 (8.40, 8.47)	9.45 (9.41, 9.49)	8.90 (8.87, 8.93)	<0.001
Breast cancer	4.64 (4.61, 4.67)	4.83 (4.80, 4.86)	4.73 (4.71, 4.75)	<0.001
Cataract	24.37 (24.32, 24.43)	25.16 (25.10, 25.22)	24.73 (24.69, 24.77)	<0.001
Chronic Kidney Disease	21.43 (21.38, 21.49)	21.10 (21.04, 21.15)	21.28 (21.24, 21.32)	<0.001
Chronic Obstructive Pulmonary Disease	14.47 (14.43, 14.52)	14.62 (14.57, 14.67)	14.54 (14.51, 14.58)	<0.001
Colorectal cancer	1.53 (1.51, 1.54)	1.58 (1.56, 1.60)	1.55 (1.54, 1.56)	<0.001
Congestive Heart Failure	19.47 (19.42, 19.52)	21.03 (20.98, 21.09)	20.18 (20.15, 20.22)	<0.001
Depression	20.34 (20.29, 20.39)	20.97 (20.92, 21.03)	20.63 (20.59, 20.67)	<0.001
Diabetes	31.29 (31.24, 31.35)	34.18 (34.11, 34.25)	32.61 (32.57, 32.65)	<0.001
Endometrial cancer	0.42 (0.42, 0.43)	0.46 (0.45, 0.47)	0.44 (0.43, 0.44)	<0.001
Glaucoma	13.43 (13.39, 13.48)	14.71 (14.66, 14.76)	14.02 (13.98, 14.05)	<0.001
Hyperlipidemia	60.38 (60.32, 60.45)	61.92 (61.86, 61.99)	61.09 (61.04, 61.13)	<0.001
Hypertension	74.43 (74.37, 74.48)	74.50 (74.44, 74.56)	74.46 (74.42, 74.50)	0.09
Hypothyroidism	21.14 (21.09, 21.19)	21.61 (21.55, 21.66)	21.35 (21.32, 21.39)	<0.001
Ischemic Heart Disease	37.78 (37.72, 37.84)	41.62 (41.55, 41.68)	39.53 (39.48, 39.57)	<0.001
Lung cancer	1.04 (1.02, 1.05)	1.06 (1.04, 1.07)	1.05 (1.04, 1.06)	0.03
Osteoporosis	11.32 (11.28, 11.36)	12.69 (12.64, 12.73)	11.94 (11.91, 11.97)	<0.001
Prostate cancer	3.96	3.96	3.96	0.75

© 2021 Thirukumaran CP et al. *JAMA Network Open.*

	Non-CJR MSAs	CJR MSAs	Total	p-value[c]
	(3.94, 3.99)	(3.93, 3.99)	(3.94, 3.98)	
Stroke/ Transient ischemic attack	5.44 (5.41, 5.47)	5.52 (5.49, 5.55)	5.48 (5.45, 5.50)	<0.001

Abbreviations: CJR: Comprehensive Care for Joint Replacement Model; MSA: Metropolitan Statistical Area; %: Column percentage; CI: Confidence Interval

Note: [a] Data from the 2013 Master Beneficiary Summary File – Base and Chronic Conditions Segment; [b] Chronic Conditions present at the end of the year as determined by the claims criteria; [c] p-values for Kruskal-Wallis tests (for continuous variables) or chi-square tests (for categorical variables) that test for the distribution of characteristics across CJR and non-CJR MSAs.

© 2021 Thirukumaran CP et al. *JAMA Network Open.*

eTable 2. Descriptive statistics for Medicare beneficiaries residing in CJR and non-CJR MSAs with a diagnosis of rheumatoid arthritis/osteoarthritis who underwent hip replacements in 2013

	Non-CJR MSAs	CJR MSAs	Total	p-value[c]
**Patients[a]**				
N	25,682	20,425	46,107	
Age in years: Mean (SD)	74.57 (6.54)	74.81 (6.65)	74.68 (6.59)	<0.001
Female: % (95% CI)	62.27 (61.67, 62.86)	62.11 (61.44, 62.78)	62.20 (61.75, 62.64)	0.73
Race: % (95% CI)				<0.001
White	95.27 (95.01, 95.53)	94.76 (94.45, 95.06)	95.05 (94.84, 95.24)	
Black	4.52 (4.27, 4.78)	4.68 (4.39, 4.97)	4.59 (4.40, 4.78)	
Hispanic	0.21 (0.15, 0.27)	0.56 (0.47, 0.68)	0.36 (0.31, 0.42)	
Dual-eligible: % (95% CI)	3.89 (3.66, 4.13)	4.41 (4.13, 4.70)	4.12 (3.94, 4.30)	0.01
Race-Dual-eligibility: % (95% CI)				<0.001
White non-dual	92.26 (91.93, 92.58)	91.53 (91.14, 91.91)	91.94 (91.68, 92.18)	
White dual	3.01 (2.81, 3.23)	3.23 (2.99, 3.48)	3.11 (2.95, 3.27)	
Black non-dual	3.71 (3.49, 3.95)	3.87 (3.61, 4.15)	3.78 (3.61, 3.96)	
Black dual	0.81 (0.70, 0.92)	0.80 (0.69, 0.94)	0.80 (0.73, 0.89)	
Hispanic non-dual	0.14 (0.09, 0.19)	0.19 (0.14, 0.26)	0.16 (0.13, 0.20)	
Hispanic dual	0.07 (0.04, 0.11)	0.37 (0.29, 0.47)	0.20 (0.16, 0.25)	
Chronic conditions[b]: % (95% CI)				
Acute myocardial infarction	0.69 (0.59, 0.79)	0.68 (0.57, 0.80)	0.68 (0.61, 0.76)	0.95
Alzheimer's disease and its related dementias	5.24 (4.97, 5.52)	5.63 (5.32, 5.96)	5.41 (5.21, 5.62)	0.06
Anemia	63.53 (62.93, 64.12)	69.66 (69.03, 70.29)	66.25 (65.81, 66.68)	<0.001
Asthma	10.32 (9.95, 10.70)	10.21 (9.80, 10.63)	10.27 (9.99, 10.55)	0.70
Atrial fibrillation	11.55 (11.16, 11.94)	11.98 (11.54, 12.43)	11.74 (11.45, 12.04)	0.15
Benign prostatic hyperplasia	12.70 (12.30, 13.11)	14.14 (13.67, 14.63)	13.34 (13.03, 13.65)	<0.001
Breast cancer	6.53 (6.23, 6.84)	6.26 (5.93, 6.60)	6.41 (6.19, 6.64)	0.24
Cataract	24.94 (24.42, 25.48)	25.65 (25.05, 26.25)	25.25 (24.86, 25.65)	0.09
Chronic Kidney Disease	17.76	17.72	17.75	0.91

© 2021 Thirukumaran CP et al. *JAMA Network Open.*

	Non-CJR MSAs	CJR MSAs	Total	p-value[c]
	(17.30, 18.24)	(17.20, 18.25)	(17.40, 18.10)	
Chronic Obstructive Pulmonary Disease	13.23 (12.82, 13.65)	12.90 (12.44, 13.37)	13.08 (12.78, 13.40)	0.30
Colorectal cancer	1.95 (1.79, 2.13)	1.95 (1.76, 2.15)	1.95 (1.83, 2.08)	0.99
Congestive Heart Failure	12.17 (11.77, 12.57)	13.15 (12.69, 13.62)	12.60 (12.30, 12.91)	0.002
Depression	22.04 (21.53, 22.55)	21.45 (20.89, 22.02)	21.78 (21.40, 22.16)	0.13
Diabetes	22.97 (22.46, 23.49)	24.65 (24.06, 25.24)	23.71 (23.33, 24.11)	<0.001
Endometrial cancer	0.85 (0.74, 0.97)	0.88 (0.76, 1.02)	0.87 (0.78, 0.95)	0.74
Glaucoma	11.93 (11.54, 12.34)	12.72 (12.27, 13.18)	12.28 (11.98, 12.59)	0.01
Hyperlipidemia	69.69 (69.12, 70.25)	70.29 (69.65, 70.91)	69.95 (69.53, 70.37)	0.16
Hypertension	80.47 (79.98, 80.95)	80.03 (79.48, 80.58)	80.28 (79.91, 80.64)	0.24
Hypothyroidism	23.65 (23.14, 24.18)	24.09 (23.51, 24.69)	23.85 (23.46, 24.24)	0.27
Ischemic Heart Disease	33.56 (32.98, 34.14)	36.70 (36.03, 37.36)	34.95 (34.51, 35.38)	<0.001
Lung cancer	0.93 (0.82, 1.06)	1.05 (0.92, 1.20)	0.99 (0.90, 1.08)	0.20
Osteoporosis	12.91 (12.50, 13.32)	13.63 (13.16, 14.10)	13.23 (12.92, 13.54)	0.02
Prostate cancer	5.64 (5.36, 5.93)	5.65 (5.34, 5.98)	5.65 (5.44, 5.86)	0.97
Stroke/ Transient ischemic attack	2.98 (2.77, 3.19)	3.37 (3.13, 3.63)	3.15 (3.00, 3.32)	0.02

Abbreviations: CJR: Comprehensive Care for Joint Replacement Model; MSA: Metropolitan Statistical Area; N: Number; %: Column percentage; SD: Standard deviation; CI: Confidence Interval

Notes: [a] Data from the 2013 Master Beneficiary Summary File – Base Segment; [b] Data from the 2013 Master Beneficiary Summary File – Chronic Conditions Segment; [c] p-values for Kruskal-Wallis tests (for continuous variables) or chi-square tests (for categorical variables) that test for the distribution of characteristics across CJR and non-CJR MSAs.

© 2021 Thirukumaran CP et al. *JAMA Network Open.*

eTable 3. Descriptive statistics for Medicare beneficiaries residing in CJR and non-CJR MSAs with a diagnosis of osteoarthritis/rheumatoid arthritis who underwent knee replacements in 2013

	Non-CJR MSA	CJR MSA	Total	p-value[c]
**Patients[a]**				
N	53,977	40,574	94,551	
Age in years: Mean (SD)	73.92 (6.07)	74.13 (6.12)	74.01 (6.09)	<0.001
Female: % (95% CI)	64.85 (64.45, 65.25)	65.63 (65.16, 66.09)	65.18 (64.88, 65.49)	0.01
Race: % (95% CI)				<0.001
White	93.79 (93.58, 93.99)	92.56 (92.30, 92.82)	93.26 (93.10, 93.42)	
Black	5.27 (5.09, 5.46)	5.69 (5.47, 5.92)	5.45 (5.31, 5.60)	
Hispanic	0.94 (0.86, 1.02)	1.74 (1.62, 1.88)	1.28 (1.21, 1.36)	
Dual-eligible: % (95% CI)	5.33 (5.15, 5.53)	6.66 (6.42, 6.91)	5.90 (5.76, 6.06)	<0.001
Race-Dual-eligibility: % (95% CI)				<0.001
White non-dual	90.03 (89.77, 90.28)	88.38 (88.06, 88.69)	89.32 (89.12, 89.51)	
White dual	3.77 (3.61, 3.93)	4.18 (3.99, 4.38)	3.95 (3.82, 4.07)	
Black non-dual	4.32 (4.15, 4.50)	4.59 (4.39, 4.79)	4.43 (4.30, 4.57)	
Black dual	0.95 (0.87, 1.04)	1.11 (1.01, 1.21)	1.02 (0.96, 1.08)	
Hispanic non-dual	0.32 (0.27, 0.37)	0.37 (0.32, 0.44)	0.34 (0.31, 0.38)	
Hispanic dual	0.62 (0.55, 0.68)	1.37 (1.26, 1.49)	0.94 (0.88, 1.00)	
Chronic conditions[b]: % (95% CI)				
Acute myocardial infarction	0.48 (0.42, 0.54)	0.48 (0.41, 0.55)	0.48 (0.44, 0.52)	1.00
Alzheimer's disease and its related dementias	4.78 (4.60, 4.97)	5.14 (4.93, 5.36)	4.94 (4.80, 5.08)	0.011
Anemia	58.95 (58.53, 59.36)	65.86 (65.39, 66.32)	61.91 (61.60, 62.22)	<0.001
Asthma	11.99 (11.72, 12.27)	11.74 (11.43, 12.06)	11.89 (11.68, 12.09)	0.24
Atrial fibrillation	11.41 (11.14, 11.68)	11.69 (11.38, 12.01)	11.53 (11.33, 11.74)	0.18
Benign prostatic hyperplasia	12.19 (11.92, 12.47)	12.91 (12.59, 13.24)	12.50 (12.29, 12.71)	0.001
Breast cancer	6.30 (6.10, 6.51)	6.45 (6.22, 6.70)	6.37 (6.21, 6.53)	0.35
Cataract	25.60 (25.23, 25.97)	26.13 (25.71, 26.57)	25.83 (25.55, 26.11)	0.06
Chronic Kidney Disease	18.22	18.57	18.37	0.17

© 2021 Thirukumaran CP et al. *JAMA Network Open.*

	Non-CJR MSA	CJR MSA	Total	p-value[c]
	(17.89, 18.55)	(18.19, 18.95)	(18.12, 18.62)	
Chronic Obstructive Pulmonary Disease	11.58 (11.31, 11.85)	11.79 (11.48, 12.11)	11.67 (11.47, 11.88)	0.32
Colorectal cancer	1.62 (1.51, 1.73)	1.56 (1.44, 1.69)	1.59 (1.51, 1.68)	0.47
Congestive Heart Failure	11.82 (11.54, 12.09)	12.92 (12.60, 13.25)	12.29 (12.08, 12.50)	<0.001
Depression	23.88 (23.52, 24.24)	24.57 (24.15, 24.99)	24.17 (23.90, 24.45)	0.01
Diabetes	29.58 (29.20, 29.97)	30.26 (29.81, 30.71)	29.87 (29.58, 30.16)	0.02
Endometrial cancer	0.82 (0.74, 0.90)	0.82 (0.73, 0.91)	0.82 (0.76, 0.88)	0.98
Glaucoma	12.43 (12.15, 12.71)	12.66 (12.34, 12.99)	12.53 (12.32, 12.74)	0.28
Hyperlipidemia	72.68 (72.30, 73.06)	73.21 (72.78, 73.64)	72.91 (72.62, 73.19)	0.07
Hypertension	84.84 (84.53, 85.14)	84.67 (84.32, 85.02)	84.77 (84.54, 84.99)	0.48
Hypothyroidism	25.30 (24.93, 25.67)	25.79 (25.36, 26.22)	25.51 (25.23, 25.79)	0.09
Ischemic Heart Disease	34.03 (33.63, 34.43)	36.83 (36.36, 37.30)	35.23 (34.93, 35.54)	<0.001
Lung cancer	0.65 (0.58, 0.72)	0.66 (0.58, 0.74)	0.65 (0.60, 0.71)	0.86
Osteoporosis	11.59 (11.32, 11.87)	12.88 (12.56, 13.21)	12.15 (11.94, 12.36)	<0.001
Prostate cancer	4.99 (4.81, 5.18)	4.49 (4.29, 4.69)	4.78 (4.64, 4.91)	<0.001
Stroke/ Transient ischemic attack	3.18 (3.03, 3.33)	3.14 (2.97, 3.32)	3.16 (3.05, 3.28)	0.75

Abbreviations: CJR: Comprehensive Care for Joint Replacement Model; MSA: Metropolitan Statistical Area; N: Number; %: Column percentage; SD: Standard deviation; CI: Confidence Interval

Notes: [a] Data from the 2013 Master Beneficiary Summary File – Base Segment; [b] Data from the 2013 Master Beneficiary Summary File – Chronic Conditions Segment; [c] p-values for Kruskal-Wallis tests (for continuous variables) or chi-square tests (for categorical variables) that test for the distribution of characteristics across CJR and non-CJR MSAs.

© 2021 Thirukumaran CP et al. *JAMA Network Open.*

eTable 4. Changes in knee replacements with CJR implementation

Panel A: Unadjusted relative changes in knee replacements with CJR implementation (estimates from Table 2; column A)

**Knee replacements**	**CJR MSAs**		
	Before	After	% change
White non-dual	2.28	2.38	4.39
White dual	0.77	0.81	5.19
Black non-dual	1.65	1.63	-1.21
Black dual	0.74	0.68	-8.11
Hispanic non-dual	1.30	1.41	8.46
Hispanic dual	0.90	1.06	17.78

Panel B: Estimated changes in knee replacement counts for Black non-dual- and dual-eligible beneficiaries

	**CJR MSA**		**Non-CJR MSA**		**Difference**
	Before	After	Before	After	
**Black non-dual-eligible**					
Population count (N)	112,851	123,141	157,085	170,417	
Adjusted probability with 95% CI (%)	2.14 (2.00, 2.27)	2.13 (1.97, 2.29)	0.97 (0.93, 1.01)	1.11 (1.05, 1.17)	
Surgery count (N)	2,412	2,619	1,519	1,890	
Difference (After – Before for each MSA treatment status group)		207		370	-163
% difference with respect to *Before* volume in CJR MSAs					-6.77%
**Black dual-eligible**					
Population count (N)	60,709	57,808	71,674	68,567	
Adjusted probability with 95% CI (%)	1.18 (1.09, 1.28)	1.11 (0.97, 1.25)	0.47 (0.44, 0.50)	0.58 (0.51, 0.64)	
Surgery count (N)	719	643	337	396	
Difference (After – Before for each MSA treatment status group)		-76		59	-134
% difference with respect to *Before* volume in CJR MSAs					-18.70%

Abbreviations: CJR: Comprehensive Care for Joint Replacement model; MSA: Metropolitan Statistical Area; N: Number; %: Percentage; CI: Confidence Interval

Note: Estimates rounded to the nearest two decimals or the whole number.

© 2021 Thirukumaran CP et al. *JAMA Network Open.*

eTable 5. Adjusted odds ratios for parallel trends test from multivariable models examining the odds of undergoing hip and knee replacement surgeries before the Comprehensive Care for Joint Replacement model was implemented (2013-2015)

	**Hip Replacement**	**Knee Replacement**
	Odds Ratio [95% CI]	*Odds Ratio [95% CI]*
**Year**		
2013	Ref	Ref
2014	1.04*** [1.02,1.06]	0.97*** [0.96,0.98]
2015	1.06*** [1.04,1.08]	0.97*** [0.95,0.98]
**MSA Treatment Status**		
Non-CJR MSA	Ref	Ref
CJR MSA	1.46*** [1.23,1.73]	1.88*** [1.68,2.10]
**Year x MSA Treatment Status**		
2014 x CJR MSA	1.00 [0.97,1.03]	1.01 [0.99,1.03]
2015 x CJR MSA	1.03* [1.00,1.06]	1.01 [0.99,1.03]
**Race-dual-eligibility**		
White non-dual	Ref	Ref
White dual	0.34*** [0.31,0.36]	0.41*** [0.39,0.43]
Black non-dual	0.50*** [0.47,0.54]	0.53*** [0.51,0.56]
Black dual	0.23*** [0.20,0.27]	0.25*** [0.23,0.27]
Hispanic non-dual	0.25*** [0.18,0.35]	0.61*** [0.52,0.71]
Hispanic dual	0.07*** [0.04,0.11]	0.59*** [0.52,0.66]
**Year x Race-dual-eligibility**		
2014 x White dual	1.06 [0.96,1.18]	0.98 [0.91,1.05]
2014 x Black non-dual	0.96 [0.87,1.05]	0.99 [0.94,1.06]
2014 x Black dual	0.92 [0.75,1.13]	1.12 [0.99,1.28]
2014 x Hispanic non-dual	0.92 [0.56,1.52]	1.38** [1.12,1.69]
2014 x Hispanic dual	2.00* [1.10,3.62]	1.05 [0.89,1.24]
2015 x White dual	1.03 [0.92,1.14]	0.95 [0.88,1.01]
2015 x Black non-dual	1.09 [1.00,1.19]	1.08* [1.02,1.15]
2015 x Black dual	1.04 [0.85,1.27]	1.1 [0.97,1.25]
2015 x Hispanic non-dual	1.14 [0.71,1.82]	1.15 [0.93,1.42]
2015 x Hispanic dual	1.47 [0.78,2.78]	1.02 [0.87,1.21]
**MSA Treatment Status x Race-dual-eligibility**		
CJR MSA x White dual	0.82*** [0.74,0.91]	0.90** [0.84,0.97]
CJR MSA x Black non-dual	1.15** [1.04,1.27]	1.19*** [1.12,1.27]
CJR MSA x Black dual	1.11 [0.90,1.36]	1.35*** [1.18,1.53]

© 2021 Thirukumaran CP et al. *JAMA Network Open.*

	**Hip Replacement**	**Knee Replacement**
	Odds Ratio [95% CI]	*Odds Ratio [95% CI]*
CJR MSA x Hispanic non-dual	1.1 [0.69,1.76]	1.03 [0.82,1.29]
CJR MSA x Hispanic dual	1.91* [1.13,3.21]	1.02 [0.88,1.17]
**Year x Treatment Status x Race-dual-eligibility**		
2014 x CJR MSA x White dual	1 [0.86,1.16]	1.08 [0.98,1.19]
2014 x CJR MSA x Black non-dual	1.01 [0.88,1.16]	0.97 [0.88,1.06]
2014 x CJR MSA x Black dual	1.27 [0.95,1.71]	0.97 [0.81,1.16]
2014 x CJR MSA x Hispanic non-dual	1.28 [0.66,2.48]	0.89 [0.66,1.21]
2014 x CJR MSA x Hispanic dual	0.49* [0.25,0.97]	0.99 [0.80,1.22]
2015 x CJR MSA x White dual	0.95 [0.82,1.11]	1.09 [0.99,1.20]
2015 x CJR MSA x Black non-dual	0.94 [0.82,1.07]	0.98 [0.90,1.07]
2015 x CJR MSA x Black dual	0.95 [0.70,1.27]	0.97 [0.81,1.17]
2015 x CJR MSA x Hispanic non-dual	1.15 [0.61,2.16]	1.11 [0.82,1.50]
2015 x CJR MSA x Hispanic dual	0.82 [0.40,1.67]	1.01 [0.82,1.25]
N (patients)	13,477,505	13,477,505
N (MSAs)	171	171
p-value (Year x MSA Treatment Status)	0.03	0.56
p-value(Year x Race-dual-eligibility)	0.07	0.004
p-value (Year x MSA Treatment Status x Race-dual-eligibility)	0.33	0.77

* p<0.05, ** p<0.01, *** p<0.001

Abbreviations: CI: Confidence interval; MSA: Metropolitan Statistical Area; CJR: Comprehensive Care for Joint Replacement model; N: Number

Notes: Adjusted odds ratios from patient-level multivariable logistic regression models with robust/sandwich estimators of variance. The models controlled for age, sex, comorbidities, calendar year, MSA fixed effects, and MSA weights.

© 2021 Thirukumaran CP et al. *JAMA Network Open.*

eTable 6. Full model estimates (adjusted odds ratios) from multivariable models examining the odds of undergoing hip or knee replacement surgeries before and after the Comprehensive Care for Joint Replacement model was implemented (2013-2017)

	**Hip Replacements**	**Knee Replacements**
	**Odds Ratio [95% CI]**	**Odds Ratio [95% CI]**
	Differential	Differential
**MSA Treatment Status**		
Non-CJR MSA	Ref	Ref
CJR MSA	1.54*** [1.33,1.79]	1.92*** [1.74,2.11][a]
**CJR Phase**		
Pre	Ref	Ref
Post	1.13*** [1.11,1.15]	1.02* [1.00,1.03]
**MSA Treatment Status x Phase**		
CJR MSA x Post	0.99 [0.96,1.02]	1.02* [1.01,1.04]
**Race-Dual**		
White non-dual	Ref	Ref
White dual	0.34*** [0.31,0.36]	0.42*** [0.40,0.44]
Black non-dual	0.50*** [0.47,0.54]	0.54*** [0.51,0.56]
Black dual	0.23*** [0.20,0.27]	0.25*** [0.23,0.27][a]
Hispanic non-dual	0.25*** [0.18,0.35]	0.61*** [0.52,0.71]
Hispanic dual	0.07*** [0.04,0.11]	0.59*** [0.53,0.66]
**MSA Treatment Status x Race-Dual**		
CJR MSA x White dual-eligible	0.82*** [0.73,0.91]	0.90** [0.84,0.96]
CJR MSA x Black non-dual-eligible	1.16** [1.05,1.28]	1.20*** [1.12,1.28]
CJR MSA x Black dual-eligible	1.11 [0.90,1.37]	1.35*** [1.19,1.54]
CJR MSA x Hispanic non-dual-eligible	1.10 [0.69,1.76]	1.03 [0.82,1.28]
CJR MSA x Hispanic dual-eligible	1.90* [1.13,3.20]	1.01 [0.87,1.16]
**CJR Phase x Race-Dual**		
Post x White dual-eligible	1.12* [1.01,1.24]	1.00 [0.93,1.06]
Post x Black non-dual-eligible	1.06 [0.97,1.15]	1.13*** [1.07,1.20]
Post x Black dual-eligible	1.09 [0.90,1.33]	1.21** [1.07,1.37]
Post x Hispanic non-dual-eligible	1.33 [0.86,2.07]	1.31** [1.07,1.60]
Post x Hispanic dual-eligible	2.27** [1.29,3.99]	1.05 [0.90,1.24]
**MSA Treatment Status x CJR Phase x Race-Dual**		
CJR MSA x Post x White dual-eligible	0.94 [0.81,1.08]	1.05 [0.96,1.16]
CJR MSA x Post x Black non-dual-eligible	0.94 [0.82,1.07]	0.84*** [0.77,0.92]
CJR MSA x Post x Black dual-eligible	1.05 [0.79,1.40]	0.74** [0.62,0.90]
CJR MSA x Post x Hispanic non-dual-eligible	0.92 [0.50,1.68]	0.88 [0.66,1.19]
CJR MSA x Post x Hispanic dual-eligible	0.49* [0.25,0.94]	1.10 [0.90,1.35]
**Age**	0.95*** [0.95,0.95]	0.94*** [0.94,0.94]
**Sex**		
Male	Ref	Ref
Female	0.97*** [0.96,0.98]	1.11*** [1.10,1.12]

© 2021 Thirukumaran CP et al. *JAMA Network Open.*

	Hip Replacements	Knee Replacements
	**Odds Ratio [95% CI]**	**Odds Ratio [95% CI]**
	Differential	Differential
**Chronic conditions**		
Acute myocardial infarction	0.71*** [0.67,0.75]	0.54*** [0.52,0.57]
Alzheimer's disease and its related dementias	0.46*** [0.45,0.47]	0.43*** [0.42,0.43]
Anemia	4.51*** [4.47,4.56]	3.82*** [3.79,3.85]
Asthma	1.31*** [1.29,1.33]	1.54*** [1.52,1.55]
Atrial fibrillation	1.07*** [1.05,1.08]	1.10*** [1.09,1.12]
Benign prostatic hyperplasia	1.52*** [1.49,1.54]	1.61*** [1.60,1.63]
Breast cancer	1.24*** [1.22,1.27]	1.27*** [1.26,1.29]
Cataract	0.93*** [0.92,0.94]	0.97*** [0.96,0.98]
Chronic Kidney Disease	0.72*** [0.71,0.72]	0.74*** [0.73,0.74]
Chronic Obstructive Pulmonary Disease	0.86*** [0.85,0.88]	0.71*** [0.70,0.72]
Colorectal cancer	1.08*** [1.04,1.11]	0.94*** [0.91,0.96]
Congestive Heart Failure	0.64*** [0.63,0.65]	0.62*** [0.61,0.62]
Depression	1.07*** [1.06,1.08]	1.18*** [1.17,1.19]
Diabetes	0.60*** [0.59,0.61]	0.78*** [0.77,0.78]
Endometrial cancer	1.56*** [1.48,1.64]	1.46*** [1.41,1.52]
Glaucoma	0.91*** [0.90,0.92]	0.96*** [0.95,0.97]
Hyperlipidemia	1.39*** [1.38,1.41]	1.51*** [1.50,1.53]
Hypertension	1.52*** [1.50,1.54]	2.01*** [1.99,2.02]
Hypothyroidism	1.05*** [1.04,1.06]	1.13*** [1.12,1.14]
Ischemic Heart Disease	0.85*** [0.85,0.86]	0.85*** [0.85,0.86]
Lung cancer	0.73*** [0.69,0.76]	0.52*** [0.50,0.55]
Osteoporosis	1.14*** [1.12,1.15]	1.03*** [1.02,1.04]
Prostate cancer	1.31*** [1.28,1.34]	1.25*** [1.23,1.27]
Stroke/ Transient ischemic attack	0.63*** [0.61,0.65]	0.62*** [0.61,0.64]
**Year**		
2013	Ref	Ref
2014	1.04*** [1.02,1.06]	0.97*** [0.96,0.98]
2015	1.05*** [1.03,1.07]	0.96*** [0.95,0.98]
**Year x MSA Treatment Status**		
2014 x CJR MSA	1.00 [0.97,1.03]	1.01 [0.99,1.03]
2015 x CJR MSA	1.03* [1.00,1.06]	1.01 [0.99,1.03]
**Year x Race-Dual**		
2014 x White dual-eligible	1.07 [0.96,1.18]	0.98 [0.92,1.05]
2014 x Black non-dual-eligible	0.96 [0.87,1.05]	0.99 [0.94,1.06]
2014 x Black dual-eligible	0.92 [0.75,1.13]	1.12 [0.99,1.28]
2014 x Hispanic non-dual-eligible	0.92 [0.56,1.52]	1.38** [1.12,1.69]
2014 x Hispanic dual-eligible	2.00* [1.10,3.62]	1.05 [0.89,1.24]
2015 x White dual-eligible	1.03 [0.93,1.14]	0.95 [0.89,1.01]
2015 x Black non-dual-eligible	1.09 [1.00,1.19]	1.08* [1.02,1.15]
2015 x Black dual-eligible	1.04 [0.85,1.27]	1.10 [0.97,1.25]
2015 x Hispanic non-dual-eligible	1.14 [0.71,1.82]	1.15 [0.93,1.42]
2015 x Hispanic dual-eligible	1.48 [0.78,2.79]	1.03 [0.87,1.22]
**Year x MSA Treatment Status x Race-Dual**		
2014 x CJR MSA x White dual-eligible	1.00 [0.86,1.16]	1.08 [0.98,1.19]

© 2021 Thirukumaran CP et al. *JAMA Network Open.*

	Hip Replacements	Knee Replacements
	**Odds Ratio [95% CI]**	**Odds Ratio [95% CI]**
	Differential	Differential
2014 x CJR MSA x Black non-dual-eligible	1.01 [0.88,1.16]	0.97 [0.88,1.06]
2014 x CJR MSA x Black dual-eligible	1.27 [0.95,1.71]	0.97 [0.81,1.17]
2014 x CJR MSA x Hispanic non-dual-eligible	1.29 [0.67,2.48]	0.89 [0.66,1.21]
2014 x CJR MSA x Hispanic dual-eligible	0.49* [0.25,0.97]	0.99 [0.80,1.22]
2015 x CJR MSA x White dual-eligible	0.95 [0.82,1.11]	1.09 [0.99,1.20]
2015 x CJR MSA x Black non-dual-eligible	0.94 [0.82,1.07]	0.98 [0.90,1.07]
2015 x CJR MSA x Black dual-eligible	0.95 [0.70,1.27]	0.97 [0.81,1.17]
2015 x CJR MSA x Hispanic non-dual-eligible	1.15 [0.62,2.16]	1.11 [0.82,1.50]
2015 x CJR MSA x Hispanic dual-eligible	0.82 [0.40,1.66]	1.01 [0.82,1.24]
N (beneficiary-year)	18,403,141	18,403,141
N (MSAs)	171	171
p-value for triple interaction term	0.28	<0.001

* p<0.05, ** p<0.01, *** p<0.001

Abbreviations: CI: Confidence interval; MSA: Metropolitan Statistical Area; CJR: Comprehensive Care for Joint Replacement model; N: Number

Notes: Adjusted odds ratios from patient-level multivariable logistic regression models with triple differences estimation and robust/sandwich estimators of variance. The models also controlled for MSA fixed effects and MSA weights. The analysis excluded data from 2016 because the CJR was introduced in April 2016, and this implementation precludes the classification of all Medicare beneficiaries into a pre- and post-cohort. Fixed effect estimates for the 171 MSAs are not presented in this table. For the year estimate and the year interaction estimates, estimates for 2017 are omitted due to collinearity.

[a] Estimates used for supporting the pre-existing disparities between White non-dual-eligible beneficiaries and Black dual-eligible beneficiaries: Probability of knee replacements White dual-eligible beneficiaries in comparison to Black non-dual-eligible beneficiaries =1/(1.92 x 0.25) = 2.08

© 2021 Thirukumaran CP et al. *JAMA Network Open.*

eTable 7. Sensitivity analyses (1-6) - the Comprehensive Care for Joint Replacement model's association with the probability of undergoing hip replacements with respect to White non-dual-eligible beneficiaries (2013-2017)

Difference in adjusted probabilities of surgeries in comparison to White non-dual-eligible beneficiaries among CJR versus non-CJR MSAs[a]								
Analysis	**Main Analysis: Triple Differences**	**Intention-to-treat analysis (SA 1)**	**RTI indicator for race (SA 2)**	**Medicare entitlement for dual-eligibility (SA 3)**	**2016 included in the post-CJR phase (SA 4)**	**Medicare's definition for elective surgeries (SA 5)**	**Race interacted with key terms (SA 6)**	**Dual interacted with key terms (SA 6)**
	%-point difference (95% CI)	*%-point difference (95% CI)*	*%-point difference (95% CI)*	*%-point difference (95% CI)*	*%-point difference (95% CI)*	*%-point difference (95% CI)*	*%-point difference (95% CI)*	*%-point difference (95% CI)*
N	18,403,141	19,875,207	18,446,093	18,403,141	23,239,775	18,411,681	18,403,141	18,403,141
**Hip Replacements**								
White dual	-0.06 (-0.14, 0.01)	-0.06 (-0.13, 0.01)	-0.08* (-0.16, -0.01)	-0.06 (-0.14, 0.02)	-0.06 (-0.13, 0.01)	-0.08* (-0.15, -0.01)		
Black non-dual	-0.05 (-0.16, 0.06)	-0.07 (-0.17, 0.03)	-0.05 (-0.16, 0.06)	-0.05 (-0.15, 0.06)	-0.05 (-0.16, 0.05)	-0.05 (-0.16, 0.05)		
Black dual	0.01 (-0.11, 0.13)	0.03 (-0.09, 0.14)	0.02 (-0.10, 0.14)	0.02 (-0.11, 0.14)	0.01 (-0.10, 0.12)	0.01 (-0.11, 0.13)		
Hispanic non-dual	-0.001 (-0.29, 0.29)	0.01 (-0.29, 0.28)	-0.07 (-0.20, 0.07)	-0.01 (-0.29, 0.26)	0.00 (-0.27, 0.27)	-0.02 (-0.31, 0.26)		
Hispanic dual	-0.14 (-0.29, 0.01)	-0.13 (-0.26, 0.001)	-0.02 (-0.12, 0.07)	-0.13 (-0.28, 0.01)	-0.12 (-0.25, 0.01)	-0.17* (-0.32, -0.02)		
Black							-0.03 (-0.12, 0.07)	
Hispanic							-0.11 (-0.32, 0.10)	
Dual								-0.05 (-0.11, 0.02)

* p<0.05, ** p<0.01, *** p<0.001

Abbreviations: MSA: Metropolitan Statistical Area; CJR: Comprehensive Care for Joint Replacement model; SA: Sensitivity analysis; RTI: Research Triangle Institute; CI: Confidence interval; N: Number; %: Percentage

© 2021 Thirukumaran CP et al. *JAMA Network Open.*

Notes: Adjusted probabilities from patient-level multivariable logistic regression models with robust/sandwich estimators of variance. The models assessed CJR's association with the probability of surgeries for each race/dual group (versus White non-dual-eligible beneficiaries) in CJR MSAs versus non-CJR MSAs. The models controlled for age, sex, comorbidities, calendar year (and interactions with CJR MSA indicator and race/dual-eligibility indicator), MSA fixed effects, and MSA weights. The probabilities and the change in probabilities were obtained using Stata's margins and lincom commands.

[a] Percentage point difference in the probability of surgeries for each race/dual group (versus White non-dual-eligible beneficiaries) in CJR MSAs with CJR implementation versus non-CJR MSAs ("triple difference").

© 2021 Thirukumaran CP et al. *JAMA Network Open.*

eTable 8. Sensitivity analyses (1-6) - the Comprehensive Care for Joint Replacement model's association with the probability of undergoing knee replacements with respect to White non-dual-eligible beneficiaries (2013-2017)

Difference in adjusted probabilities of surgeries in comparison to White non-dual-eligible beneficiaries among CJR versus non-CJR MSAs[a]								
Analysis	**Main Analysis: Triple Differences**	**Intention-to-treat analysis (SA 1)**	**RTI indicator for race (SA 2)**	**Medicare entitlement for dual-eligibility (SA 3)**	**2016 included in the post-CJR phase (SA 4)**	**Medicare's definition for elective surgeries (SA 5)**	**Race interacted with key terms (SA 6)**	**Dual interacted with key terms (SA 6)**
	%-point difference (95% CI)	*%-point difference (95% CI)*	*%-point difference (95% CI)*	*%-point difference (95% CI)*	*%-point difference (95% CI)*	*%-point difference (95% CI)*	*%-point difference (95% CI)*	*%-point difference (95% CI)*
N	18,403,141	19,875,207	18,446,093	18,403,141	23,239,775	18,411,681	18,403,141	18,403,141
**Knee Replacements**								
White dual	0.01 (-0.10, 0.12)	0.02 (-0.09, 0.12)	-0.03 (-0.15, 0.09)	0.02 (-0.10, 0.13)	0.01 (-0.10, 0.12)	-0.02 (-0.13, 0.10)		
Black non-dual	-0.25** (-0.40, -0.10)	-0.21** (-0.35, -0.07)	-0.24** (-0.39, -0.09)	-0.24** (-0.38, -0.09)	-0.24** (-0.39, -0.09)	-0.25* (-0.40, -0.10)		
Black dual	-0.27** (-0.45, -0.10)	-0.24* (-0.40, -0.07)	-0.30* (-0.47, -0.13)	-0.31* (-0.48, -0.13)	-0.27* (-0.44, -0.10)	-0.28* (-0.45, -0.11)		
Hispanic non-dual	-0.04 (-0.65, 0.57)	-0.12 (-0.70, 0.46)	-0.01 (-0.25, 0.24)	-0.01 (-0.60, 0.58)	-0.04 (-0.62, 0.53)	-0.03 (-0.64, 0.58)		
Hispanic dual	0.23 (-0.10, 0.55)	0.33* (0.02, 0.64)	0.29* (0.07, 0.51)	0.21 (-0.12, 0.54)	0.22 (-0.09, 0.53)	0.25 (-0.07, 0.58)		
Black							-0.28*** (-0.41, -0.15)	
Hispanic							0.13 (-0.29, 0.55)	
Dual								-0.02 (-0.12, 0.08)

* p<0.05, ** p<0.01, *** p<0.001

Abbreviations: MSA: Metropolitan Statistical Area; CJR: Comprehensive Care for Joint Replacement model; SA: Sensitivity analysis; RTI: Research Triangle Institute; CI: Confidence interval; N: Number; %: Percentage

© 2021 Thirukumaran CP et al. *JAMA Network Open.*

Notes: Adjusted probabilities from patient-level multivariable logistic regression models with robust/sandwich estimators of variance. The models assessed CJR's association with the probability of surgeries for each race/dual group (versus White non-dual-eligible beneficiaries) in CJR MSAs versus non-CJR MSAs. The models controlled for age, sex, comorbidities, calendar year (and interactions with CJR MSA indicator and race/dual-eligibility indicator), MSA fixed effects, and MSA weights. The probabilities and the change in probabilities were obtained using Stata's margins and lincom commands.

[a] Percentage point difference in the probability of surgeries for each race/dual group (versus White non-dual-eligible beneficiaries) in CJR MSAs with CJR implementation versus non-CJR MSAs ("triple difference").

© 2021 Thirukumaran CP et al. *JAMA Network Open.*

eTable 9. Sensitivity analysis (7) – the Comprehensive Care for Joint Replacement model's effect on the probability of undergoing surgery in the MSA of residence

**Difference in the adjusted probabilities of undergoing surgeries in MSA of residence with CJR implementation**		
	**Hip Replacement**	**Knee Replacement**
	%-point difference (95% CI)	*%-point difference (95% CI)*
N	90,935	173,310
White non-dual	0.44 (-0.05, 0.92)	-0.46* (-0.82, -0.10)
White dual	0.14 (-1.90, 2.18)	-1.42* (-2.79, -0.04)
Black non-dual	0.79 (-0.90, 2.48)	0.32 (-0.73, 1.37)
Black dual	0.22 (-3.52, 3.95)	0.11 (-2.22, 2.44)
Hispanic non-dual	3.79 (-3.58, 11.17)	-0.42 (-4.10, 3.26)
Hispanic dual	-0.64 (-5.86, 4.59)	0.54 (-1.21, 2.29)

* $p<0.05$, ** $p<0.01$, *** $p<0.001$

Abbreviations: CJR: Comprehensive Care for Joint Replacement model; MSA: Metropolitan Statistical Area; CI: Confidence interval; N: Number; %: Percentage

Notes: Cohort is limited to beneficiaries residing in CJR MSAs. Adjusted probabilities from patient-level multivariable logistic regression models with robust/sandwich estimators of variance. Outcome was a binary indicator of whether the patient underwent surgery in the MSA of residence among CJR MSAs. The key independent variables were the CJR phase, the race/dual-eligibility indicator, and the interaction between these variables. The models controlled for age, sex, comorbidities, calendar year, MSA fixed effects, and MSA weights. The differences in probabilities were obtained using Stata's lincom command.

© 2021 Thirukumaran CP et al. *JAMA Network Open.*

eReferences

1. Research Data Assistance Center (ResDAC). MedPAR RIF. https://www.resdac.org/cms-data/files/medpar. Accessed 03/15, 2021.

2. Barnett ML, Wilcock A, McWilliams JM, et al. Two-year evaluation of mandatory bundled payments for joint replacement. *N Engl J Med*. 2019;380(3):252-262.

3. Rudasill SE, Dattilo JR, Liu J, Kamath AF. Hemiarthroplasty or Total Hip Arthroplasty: Is There a Racial Bias in Treatment Selection for Femoral Neck Fractures?. *Geriatric orthopaedic surgery & rehabilitation*. 2019;10:2151459319841741.

4. Centers for Medicare and Medicaid Services. Bundled Payments for Care Improvement. https://innovation.cms.gov/innovation-models/bundled-payments. Accessed 03/15, 2021.

5. Chronic Condition Data Warehouse. Options for determining which CMS Medicare beneficiaries are dually eligible for Medicare and Medicaid benefits–A technical guidance paper. https://www.resdac.org/sites/resdac.umn.edu/files/ccw-technical-guidance-options-for-determining-dually-eligibles.pdf. Accessed 03/15, 2021.

6. Ryan AM, Kontopantelis E, Linden A, Burgess Jr JF. Now trending: Coping with non-parallel trends in difference-in-differences analysis. *Stat Methods Med Res*. 2019;28(12):3697-3711.

7. Dimick JB, Nicholas LH, Ryan AM, Thumma JR, Birkmeyer JD. Bariatric surgery complications before vs after implementation of a national policy restricting coverage to centers of excellence. *JAMA*. 2013;309(8):792-799.

8. Kahn JM, Davis BS, Yabes JG, et al. Association between state-mandated protocolized sepsis care and in-hospital mortality among adults with sepsis. *JAMA*. 2019;322(3):240-250.

9. Jarrín OF, Nyandege AN, Grafova IB, Dong X, Lin H. Validity of Race and Ethnicity Codes in Medicare Administrative Data Compared With Gold-standard Self-reported Race Collected During Routine Home Health Care Visits. *Med Care*. 2020;58(1):e1-e8.

10. Eicheldinger C, Bonito A. More accurate racial and ethnic codes for Medicare administrative data. *Health Care Financ Rev*. 2008;29(3):27.

11. Centers for Medicare and Medicaid Services. 2019 Procedure-Specific Complication Measure Updates and Specifications: THA/TKA. https://www.qualitynet.org/dcs/ContentServer?c=Page&pagename=QnetPublic%2FPage%2FQnetTier4&cid=1228772782693. Accessed 03/15, 2021.

12. Centers for Medicare and Medicaid Services. 2019 Procedure-Specific Readmission Measure Updates and Specification Report: Total Hip Arthroplasty and/or Total Knee Arthroplasty and Coronary Artery Bypass Graft. https://www.qualitynet.org/dcs/ContentServer?c=Page&pagename=QnetPublic%2FPage%2FQnetTier4&cid=1219069855841. Accessed 03/15, 2021.

13. The Lewin Group Inc. CMS Comprehensive Care for Joint Replacement Model: Performance Year 2 Evaluation Report - Appendices. https://downloads.cms.gov/files/cmmi/cjr-secondannrpt-app.pdf. Accessed 03/15, 2021.

14. StataCorp L. Stata 16 help for weight. https://www.stata.com/help16.cgi?weight. Accessed 03/15, 2021.

© 2021 Thirukumaran CP et al. *JAMA Network Open.*
